# Efficacy and influencing factors of modified electroconvulsive therapy for schizophrenia: a real-world retrospective observational study

**DOI:** 10.3389/fpsyt.2025.1654151

**Published:** 2025-10-03

**Authors:** Zhiping Wang, Jiancheng Qiu, Ping Zhang, Minmin Chen, Xueting Wang

**Affiliations:** Nantong Mental Health Center (The Fourth People's Hospital of Nantong), Nangtong, Jiangsu, China

**Keywords:** schizophrenia, SCZ, MECT, electroconvulsive therapy, EEG seizure duration, PANSS, treatment predictors, personalised psychiatry

## Abstract

**Background:**

Schizophrenia (SCZ) is a chronic and disabling psychiatric disorder. Modified Electroconvulsive Therapy (MECT), which involves electrical stimulation under general anaesthesia and muscle relaxation, is widely used to treat SCZ. Despite its rapid onset and robust therapeutic effect, the efficacy of MECT varies significantly between individuals. This study aimed to evaluate the clinical effectiveness of MECT in patients with SCZ and identify its influencing factors, with the goal of informing personalised treatment strategies.

**Methods:**

This retrospective observational study included 237 inpatients with SCZ who received a full course of MECT at the Fourth People’s Hospital of Nantong between January 2023 and December 2024. Treatment response was evaluated using the Positive and Negative Syndrome Scale (PANSS) reduction rate. Patients were classified into effective (≥50% reduction) and ineffective (<50% reduction) groups. Demographic, clinical, and treatment-related variables were compared between groups, and multivariate logistic regression was used to identify predictors of treatment response.

**Results:**

The overall effectiveness rate of MECT was 70.46%. Multivariate analysis identified age ≥50 years (OR = 0.111–0.078, P = 0.010–0.002) and illness duration ≥10 years (OR = 0.028–0.003, P < 0.05) as negative predictors of response. In contrast, first-episode SCZ (OR=6.537, P = 0.003), higher baseline positive symptom scores (OR = 1.325, P<0.001), and longer EEG seizure duration (OR = 1.183, P<0.001) were positive predictors. No significant associations were found for sex, education level, or stimulus parameters such as current or frequency.

**Conclusion:**

MECT remains a clinically valuable intervention for SCZ, particularly in younger, first-episode patients with prominent positive symptoms. Treatment efficacy is influenced by age, illness duration, baseline symptom severity, and seizure quality. These findings support the need for personalised MECT protocols guided by clinical and electrophysiological characteristics.

## 1 Introduction

Schizophrenia (SCZ) is a chronic and disabling psychiatric disorder characterised by symptoms including positive symptoms (such as hallucinations, delusions, and disorganised thinking), negative symptoms (such as affective flattening and social withdrawal), and behavioural disturbances (1). Typically manifesting in adolescence or early adulthood, SCZ has become one of the global causes of disability (1, 2). Although antipsychotics have significantly improved clinical outcomes of patients (3), their adverse effects—particularly metabolic syndrome and extrapyramidal symptoms—remain a major clinical challenge (4). In this context, physical interventions have attention, and Modified Electroconvulsive Therapy (MECT) as a promising therapeutic approach owing to its unique mechanism of action (5, 6).

MECT involves the induction of brief, generalised cerebral seizures under anaesthesia, which may contribute to rebalancing neurotransmitter systems and modulating functional brain networks (7, 8). Existing evidence supports the efficacy of MECT in the treatment of SCZ episodes, agitation, and catatonia (8–11), and it is particularly beneficial for patients who are treatment-resistant or require urgent clinical intervention (12–15). Furthermore, several studies have demonstrated that MECT can significantly reduce the length of hospital stay in patients with schizophrenia, thereby alleviating the burden on families and healthcare systems (16–18).

Although previous studies have investigated individual predictors of MECT efficacy—for example patient demographics (age, illness duration), clinical measures (Positive and Negative Syndrome Scale scores) or single treatment parameters (stimulus intensity) (19, 20)—there is a clear lack of comprehensive, multivariable investigations that integrate these domains and examine their joint, potentially interacting effects. For instance, one influential study reported that bitemporal stimulus intensities above 1.5× the seizure threshold may accelerate improvement in positive symptoms, but it did not account for clinical or demographic covariates that could modify this relation (19).To address this evidence gap, the present retrospective study systematically evaluates how demographic characteristics, clinical phenotype and detailed MECT parameters jointly relate to symptomatic outcomes in patients with schizophrenia. Our objective is to generate robust, multivariable evidence to inform the optimisation and individualisation of MECT protocols in routine clinical practice.

## 2 Materials and methods

### 2.1 Participants

**Figure 1:**
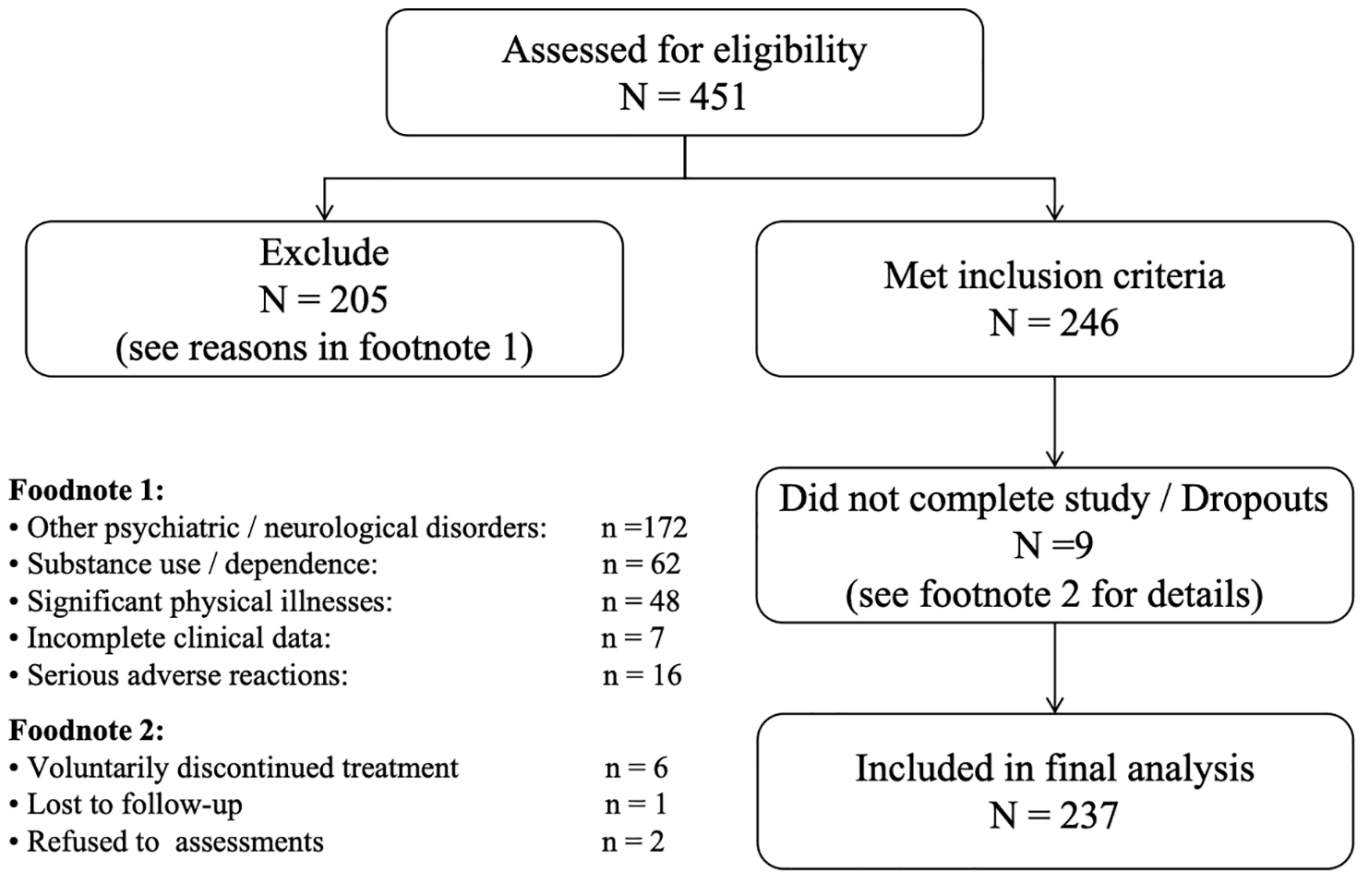
STROBE flow diagram of patient screening, exclusions, and inclusion in the final analysis.

All consecutive adult in-patients diagnosed with schizophrenia at the Fourth People’s Hospital of Nantong between January 2023 and December 2024 were screened for eligibility (n = 451). Of these, 205 patients were excluded because they met one or more exclusion criteria; note that category counts are not mutually exclusive and therefore do not sum to the overall exclusion total. 9 patients were not evaluable in the primary analysis owing to dropout. The remaining 237 patients comprised the analysed sample (see **Figure 1** for full details).

1. Inclusion criteria: Patients who (a) were aged 18 - 65 years; (b) met the diagnostic criteria for SCZ according to either the Diagnostic and Statistical Manual of Mental Disorders, Fifth Edition or the International Classification of Diseases, Tenth Revision (21); (c) han no contraindications; (d) voluntarily accepted MECT and had not received MECT within the past six months; (e) completed ≥6 MECT sessions with complete pre- and post-treatment evaluation data; and (f) experienced no serious adverse events requiring treatment discontinuation occurred during the course.
2. Exclusion criteria: Patients with (a) other psychiatric disorders (e.g., bipolar disorder or major depressive episode) or neurological disorders (e.g. bipolar disorder, epilepsy); (b) substance use or drug dependence; (c) significant physical illnesses;(d) incomplete clinical data; or (e) serious adverse reactions to MECT.
3. Drop-out criteria: Patients who **(a)** voluntarily discontinued treatment; (b) lost to follow-up; or (c) who refused to complete post-treatment assessments.

### 2.2 Treatment procedure

All procedures were conducted in strict accordance with the Expert Consensus on Modified Electroconvulsive Therapy (2019 Edition) (7). Treatment was administered using a Thymatron IV device (Somatics, Lake Bluff, IL, USA). Pre-treatment protocols included fasting and water restriction, routine screening with Electrocardiograph, chest X-ray, and blood tests to exclude contraindications.

During treatment, vital signs were continuously monitored. Anaesthesia was induced using etomidate (0.16–0.2 mg/kg), muscle relaxation was achieved with succinylcholine (1.0 mg/kg), and atropine sulphate (0.01 mL/kg) was used to reduce airway secretions. Bilateral temporal electrodes were used to induce seizures (22). Initial stimulus dosing was calculated using an age-based formula, with subsequent adjustments based on seizure adequacy assessed by electroencephalographic (EEG) monitoring (18), as detailed in Supplementary 1 Seizure adequacy criteria and dose−titration algorithm.

MECT was administered three times during the first week (on alternate days) and twice weekly during the second week, resulting in a total of six sessions over two weeks. Each treatment was jointly supervised by an anaesthesiologist and a psychiatrist throughout the procedure (18).

### 2.3 Outcome assessment and grouping

The Positive and Negative Syndrome Scale (PANSS) was used to evaluate clinical efficacy. PANSS comprises three subscales: positive symptoms (7 items), negative symptoms (7 items), and general psychopathology (16 items), with total scores ranging from 30 to 210. Assessments were conducted at baseline (within 24 hours before the first MECT session) and post-treatment (within 24 hours after the final session).

Efficacy was determined based on the reduction rate of the PANSS total score, calculated as:

[(Baseline score − Post-treatment score)/Baseline score] × 100% (23).

We chose a ≥50% reduction in total PANSS score to define treatment response for three principal reasons. First, a 50% reduction is widely regarded in the MECT literature as representing a clinically meaningful improvement and is commonly used in studies that classify outcomes as “markedly effective” (≥75%), “effective” (50–74%), “improved” (25–49%) and “ineffective” (<25%) (24). This categorical scheme has precedent in prior MECT reports (11, 25, 26), facilitating direct comparability with the existing body of MECT evidence. Second, empirical work linking PANSS percentage change to the Clinical Global Impression (CGI) suggests that reductions in the order of ~40–50% correspond to CGI categories such as “much improved”, whereas smaller reductions of 20–30% typically reflect only minimal-to-moderate clinical change (27). Thus, a 50% threshold better captures treatment effects that are likely to be meaningful to clinicians and patients. Third, selecting ≥50% strikes a pragmatic balance between sensitivity and specificity in a treatment context where rapid and substantial symptomatic relief (rather than marginal change) is the clinical aim.

### 2.4 Observational variables

Demographic characteristics included age, sex, and education. Health-related variables comprised smoking, alcohol consumption, family history of mental illness, and body mass index (BMI). Smoking status was dichotomised based on history (smoker vs non-smoker). Alcohol use was assessed in accordance with WHO guidelines (Global Status Report on Alcohol and Health), categorised as non-drinker, moderate drinker, or harmful drinker. Harmful drinking was defined as a daily average alcohol intake of ≥61g for men and ≥41g for women.

Clinical characteristics included illness duration, first-episode status, and antipsychotic medication dose converted to olanzapine equivalents (28, 29) (see **Supplementary Table S1**). Baseline scores for PANSS positive, negative, general psychopathology, and total score were also collected.

MECT treatment parameters included EEG seizure duration (30), Average Seizure Energy Index (ASEI), Postictal Suppression Index (PSI), static and dynamic impedance, energy percentage, stimulus dose, current, frequency, and duration. The ASEI represents the averaged ictal EEG power, reflecting the overall “potency” of the seizure, and is calculated over the entire seizure duration. The PSI quantifies the proportion of abrupt termination of ictal activity versus a gradual, undifferentiated decline. Dynamic impedance is measured automatically by the Thymatron during stimulation, whereas static impedance whereas static impedance was measured after EEG electrode placement and prior to stimulation; both values are expressed in ohms (Ω).These definitions and calculations follow the Thymatron IV manual.

### 2.5 Assessment and quality control

All clinical evaluations were independently conducted by two psychiatrists with attending-level qualifications and formal training in PANSS assessment. Baseline assessments were conducted within 24 hours before MECT initiation, and post-treatment assessments were completed within 24 hours following the final session. Interviews using a standardised protocol.

Data were entered in real-time into an electronic database and double-checked by an independent data manager using a blinded approach. Ten percent of the sample was randomly selected for duplicate data entry verification before database locking.

### 2.6 Statistical analysis

Statistical analyses were performed STATA 16.0 (StataCorp LLC, USA). Continuous variables were inspected for normality using the Shapiro–Wilk test. Variables with approximately normal distributions are reported as mean ± standard deviation (SD) and compared using independent-samples t-tests. Variables with non-normal distributions are reported as median, interquartile range (IQR) and compared using the two-sided Mann–Whitney U test. Categorical variables are presented as counts (percentages) and compared by Pearson’s χ² test.

A multivariable logistic regression model was used to explore predictors of MECT efficacy. To address potential multicollinearity among predictors, Variance inflation factor (VIF) analysis was performed; variables with VIF > 5 were considered to exhibit multicollinearity. A two-tailed p-value of <0.05 was considered statistically significant.

Sensitivity analyses (1) a worst-case (intention-to-treat) sensitivity analysis in which patients who dropout (discontinued treatment or lacked post-treatment PANSS) were assumed to be non-responders; and (2) Using alternative responder thresholds ≥30%, Results from these sensitivity analyses are reported in the **Supplementary Material**. Robustness Analyses (1) Ordinal logistic model using the four pre-specified categories (≥75%, 50–74%, 25–49%, <25%); (2) Using PANSS total in place of subscales to test robustness; (3) Using beta regression to test robustness. All primary conclusions were robust to these alternative specifications.

### 2.7 Ethical statement

This study adhered to the principles of informed consent, autonomy, confidentiality, and beneficence. Written informed consent was obtained from all participants or their legal guardians, after clear explanation of the study’s objectives and procedures. Participation was voluntary and participants’ data were anonymised. The study protocol was reviewed and approved by the Ethics Committee of the Fourth People’s Hospital of Nantong City (Approval No. 2023-Ko37).

## 3 Results

Based on the percentage reduction in PANSS total scores, participants achieving a reduction rate of ≥50% (i.e. markedly effective or effective) were classified into the effective group (n = 167, 70.46%), while the remaining participants were assigned to the ineffective group (n = 70, 29.54%).

### 3.1 Comparison of baseline characteristics between groups

A comparison of demographic, health-related, and clinical characteristics between the effective and ineffective groups is presented in **Table 1**. The two groups were similar across most sociodemographic and clinical variables. The baseline differences of statistical significance were patient age distribution and first-episode status: a substantially greater proportion of non-responders were aged ≥50 years, whereas first-episode patients were more common among responders (both p < 0.05). All other baseline variables (sex, education, residence, BMI, family history, smoking and alcohol use, antipsychotic dose, and most illness-related categories) did not differ between groups (see **Table 1**).

**Table 1:** Comparison of demographic and clinical characteristics between effective and ineffective groups.

Variable	Ineffective group (n=70)	Effective group (n=167)	*x^2^/t/z*-value	*p*-value
Age group (years, %)				
<30	7(10.00)	35(20.96)	54.524	<0.001
30-40	10(14.29)	55(32.93)		
40-50	12(17.14)	56(33.53)		
50-60	20(28.57)	12(7.19)		
≥60	21(30.00)	9(5.39)		
Sex(%)				
Male	35(50.00)	77(46.11)	0.300	0.584
Female	35(50.00)	90(53.89)		
Education level(%)				
Less than lower secondary	53(75.71)	131(78.44)	1.643	0.440
upper secondary & vocational training	9(12.86)	25(14.97)		
tertiary	8(11.43)	11(6.59)		
Marital status(%)				
With partner	6(8.57)	24(14.37)	1.501	0.221
Single	64(91.43)	143(85.63)		
Residence(%)				
Urban	38(54.29)	106(63.47)	1.746	0.186
Rural	32(45.71)	61(36.53)		
BMI(kg/m², mean ± SD)	25.48 ± 6.76	26.53 ± 3.91	-1.490	0.138
Family history of SCZ(%)				
No	63(90.00)	139(83.23)	1.794	0.180
Yes	7(10.00)	28(16.77)		
Smoking(%)				
No	49(70.00)	111(66.47)	0.281	0.596
Yes	21(30.00)	56(33.53)		
Alcohol use(%)				
Non-drinker	50(71.43)	113(67.66)	1.786	0.409
Moderate drinking	9(12.86)	16(9.58)		
Harmful drinking	11(15.71)	38(22.75)		
Antipsychotic dose [mg/d, median(IQR)]	13.17 (10.06, 17.78)	13.15 (8.27, 18.36)	0.444	0.657
Illness duration(years, %)				
	6(8.57)	17(10.18)	0.898	0.826
5-10	6(8.57)	15(8.98)		
10-15	8(11.43)	13(7.78)		
≥15	50(71.43)	122(73.05)		
First-episode status(%)				
No	54(77.14)	97(58.08)	7.750	0.005
Yes	16(22.86)	70(41.92)		

### 3.2 Comparison of MECT treatment parameters between groups.

The principal electrophysiological difference between groups was EEG seizure duration: responders had significantly longer than non-responders (p < 0.001). No other procedural or device parameters showed differences in comparisons (**Table 2**).

**Table 2:** Comparison of MECT parameters between effective and ineffective groups.

Parameter	Ineffective group	Effective group	*t/z*-value	*p*-value
	(n = 70)	(n = 167)		
EEG seizure duration [s, median(IQR)]	39(29,51)	52(41,61)	-4.241	<0.001
ASEI [μV^2^, median(IQR)]	41102.05 (35360.80,45555.00)	38844.20 (33714.90,45547.20)	1.314	0.189
PSI (%)	74.25 ± 9.00	74.10 ± 9.94	0.115	0.909
Static Impedance [Ω, median(IQR)]	1051.54 (849.28,1278.49)	1085.51 (879.73,1268.91)	-0.224	0.823
Dynamic Impedance [Ω, median(IQR)]	233.84 (199.51,268.47)	221.84 (191.78,251.18)	1.910	0.056
Energy Percentage [%, median(IQR)]	69.13 (58.88,76.26)	65.72 (56.45,73.79)	1.588	0.112
Stimulus Charge [mC, median(IQR)]	299.39 (254.60,349.91)	303.27 (258.24,344.92)	0.076	0.940
Stimulus Current (A)	0.81 ± 0.06	0.82 ± 0.07	–0.724	0.470
Stimulus Frequency [Hz, median(IQR)]	57.53 (50.57,64.22)	56.52 (49.71,62.47)	1.020	0.308
Stimulus Duration [s, median(IQR)]	3.11 (2.50,3.52)	2.91 (2.40,3.39)	1.111	0.267

### 3.3 Comparison of PANSS reduction rates between groups before and after treatment.

At baseline, the effective group had higher PANSS positive and total scores. After the MECT course, responders exhibited substantially greater reductions in positive symptoms, general psychopathology and total PANSS score (all p < 0.001). There was no significant between-group difference in negative-symptom reduction (**Table 3**).

**Table 3:** Comparison of PANSS scores and reduction rates between groups.

PANSS domain	Ineffective group (n = 70)	Effective group (n = 167)	t*/z*-value	p-value
Pre-treatment PANSS scores				
Negative symptoms [median(IQR)]	26(22,29)	27(24,30)	-1.601	0.110
Positive symptoms [median(IQR)]	28(25,31)	36(29,39)	-6.344	<0.001
General psychopathology	50.56 ± 8.81	52.77 ± 8.22	–1.853	0.065
Total PANSS score	105.73 ± 12.45	112.58 ± 15.58	–3.267	0.001
Post-treatment PANSS reduction rates				
Negative symptom reduction [%, median(IQR)]	33.06(14.44,48.02)	35.33(18.84,47.44)	0.144	0.885
Positive symptom reduction [%, median(IQR)]	26.61(15.52,42.07)	70.07(62.84,75.85)	-11.742	<0.001
General psychopathology reduction [%, median(IQR)]	30.22(7.05,56.42)	53.72(44.91,61.81)	-6.130	<0.001
Total PANSS reduction [%, median(IQR)]	31.17(19.25,43.85)	52.57(51.20,54.55)	-12.139	<0.001

### 3.4 Multivariate logistic regression analysis

We fitted a multivariable logistic regression to identify independent predictors of achieving ≥50% PANSS reduction. After addressing severe multicollinearity among candidate predictors *(*see **Supplementary Materials S3** Methods for VIF-based variable selection*)*, the model retained clinical and electrophysiological covariates. Key independent predictors were: older age (≥50 years) and longer illness duration (≥10 years) - both associated with a lower likelihood of being a responder - and first-episode status, higher baseline positive-symptom severity, and longer EEG seizure duration, which were associated with increased odds of response (all P < 0.05), as shown in **Table 4**.

### 3.5 Sensitivity and robustness analysis

To test the robustness of these conclusions we (a) inspected VIFs and removed variables with extreme collinearity prior to reporting the final model (**Supplementary Table S2**), and (b) report sensitivity analyses (alternative response thresholds, continuous outcome modelling and worst-case imputation for dropouts) in the **Supplementary Material S4**. These supplementary analyses are included to address information-loss concerns inherent to dichotomisation and to verify that the identified direction of associations is robust to reasonable variations in modelling choices.

### 3.6 Adverse events

Among 237 patients (1,422 MECT sessions), no serious adverse events (AE) occurred that required permanent discontinuation. Overall, 68 patients (28.7%) experienced at least one non-serious AE. The commonest events were headache (n = 40; 16.9%), transient confusion/delirium (n = 18; 7.6%), and transient memory complaints (n = 12; 5.1%). The majority (82%) of events were mild and resolved within 72 hours with symptomatic treatment; 12% were moderate and required brief pharmacological therapy or extended observation. There were no statistically significant differences in overall AE incidence between the effective and ineffective groups (29.3% vs 27.1%, p = 0.55). Full AE details are provided in **Supplementary Materials S5**.

## 4 Discussion

This study confirmed a response rate of 70.46% for MECT in the treatment of SCZ, which aligns with previously reported ranging from 55.5% to 76.7% in both domestic and international literature (15, 23, 31). These findings further support the clinical value of MECT as an effective intervention for SCZ. Multivariate logistic regression identified age, duration of illness, first-episode status, severity of positive symptoms, and EEG seizure duration as predictors of treatment outcome, offering an evidence-based foundation for individualised treatment optimisation.

### 4.1 Demographic predictors of treatment response

Age ≥50 years emerged as a negative predictor of MECT efficacy (OR = 0.111–0.078), with younger patients demonstrating significantly greater symptomatic improvement—consistent with existing findings (32–35). These findings support a model of age-related responsiveness, suggesting that stimulation dosage should be adjusted by age, with older patients potentially requiring higher stimulus intensities to induce adequate therapeutic seizures. There is a clear need for age-adjusted dose–response algorithms to be established in future protocols.

Additionally, illness duration ≥10 years significantly reduced treatment efficacy (OR = 0.028–0.003), a result in line with previous studies (36). Chronicity in SCZ may decrease neuronal responsiveness to MECT. Notably, first-episode patients showed superior outcomes (OR = 6.537), likely due to lower seizure thresholds and higher neuroplasticity observed in early-onset patients (32). This supports the concept of a “critical treatment window,” highlighting the potential for early intervention to exert amplified effects and improve long-term outcomes (37).

Other demographic characteristics—including sex, education level, smoking and alcohol use, and family history of SCZ - were not independently associated with treatment efficacy (P > 0.05). While this finding is consistent with several prior reports (38–40), it is worth noting that one study found women to have lower seizure thresholds and more complete therapeutic seizures (41). These inconsistencies underscore the necessity of large-scale, multicentre randomised trials to determine sex-specific treatment responses and establish standardised evaluation frameworks.

### 4.2 Clinical characteristics and treatment response

MECT demonstrated substantial efficacy in reducing PANSS positive symptom scores, general psychopathology scores, and overall total scores. Although responders showed significantly greater post-treatment reductions in general psychopathology scores (**Table 3**), baseline general-psychopathology was not an independent predictor of achieving ≥50% PANSS reduction in our multivariable model. While higher baseline positive symptom scores were positively correlated with greater treatment response (OR = 1.325, p < 0.001), which was consistent with previous studies and may be involved in the recovery of cerebellar-cerebral connectivity following MECT (42).

**Table 4:** Multivariate logistic regression analysis of factors associated with MECT treatment response.

Variable	OR	SE	z	p-value	95% CI
Age group					
<30 (Ref.)	–	–	–	–	–
30–40	1.209	0.875	0.260	0.793	0.293 - 4.991
40–50	1.057	0.801	0.070	0.942	0.239 - 4.669
50–60	0.111	0.095	-2.560	0.010	0.021 - 0.597
≥60	0.078	0.065	-3.050	0.002	0.015 - 0.402
Sex					
Male (Ref.)	–	–	–	–	–
Female	2.763	1.824	1.540	0.124	0.757 - 10.078
Education level					
Less than lower secondary	–	–	–	–	–
upper secondary & vocational training	0.993	0.832	-0.010	0.993	0.192 - 5.129
tertiary	0.527	0.462	-0.730	0.465	0.095 - 2.937
Marital status					
With partner (Ref.)	–	–	–	–	–
Single	0.725	0.538	-0.430	0.665	0.169 - 3.109
Residence					
Urban (Ref.)	–	–	–	–	–
Rural	0.579	0.300	-1.050	0.292	0.209 - 1.599
BMI	1.059	0.056	1.090	0.274	0.955 - 1.175
Family history of SCZ					
No (Ref.)	–	–	–	–	–
Yes	3.113	2.556	1.380	0.166	0.623 - 15.557
Smoking					
No (Ref.)	–	–	–	–	–
Yes	1.793	1.221	0.860	0.391	0.472 - 6.814
Alcohol use					
Non-drinker (Ref.)	–	–	–	–	–
Moderate drinking	3.689	3.097	1.560	0.120	0.712 - 19.116
Harmful drinking	1.808	1.304	0.820	0.411	0.440 - 7.429
Antipsychotic Dose	0.983	0.035	-0.500	0.619	0.917 - 1.053
Illness duration					
<5 years (Ref.)	–	–	–	–	–
5–10 years	0.193	0.205	-1.550	0.122	0.024 - 1.550
10–15 years	0.028	0.036	-2.840	0.004	0.002 - 0.331
≥15 years	0.003	0.004	-4.120	<0.001	0.001 - 0.047
First-episode status					
No (Ref.)	–	–	–	–	–
Yes	6.537	4.060	3.020	0.003	1.935 - 22.083
MECT parameters					
EEG Seizure Duration	1.183	0.044	4.550	<0.001	1.100 - 1.272
ASEI*	0.202	0.226	-1.430	0.153	0.023 - 1.809
PSI	0.963	0.026	-1.400	0.162	0.914 - 1.015
Energy Percentage	1.005	0.020	0.270	0.787	0.966 - 1.046
Stimulus Current*	0.210	0.470	-0.700	0.486	0.003 - 17.005
Stimulus Duration	0.925	0.352	-0.210	0.837	0.439 - 1.949
Pre-treatment PANSS scores					
Negative symptoms	0.990	0.051	-0.190	0.848	0.896 - 1.094
Positive symptoms	1.325	0.071	5.240	<0.001	1.193 - 1.472
General psychopathology	0.960	0.027	-1.440	0.150	0.908 - 1.015

OR, Odds Ratio; SE, Standard Error; CI, Confidence Interval; BMI, Body Mass Index; EEG, Electroencephalogram; ASEI, Average Seizure Energy Index; PSI, Postictal Suppression Index.

*ASEI was log transformed and Stimulus Current was reciprocals transformed.

### 4.3 Technical parameters and electrophysiological predictors

This study explored the association between MECT technical parameters and therapeutic efficacy in SCZ, identifying EEG seizure duration as a key electrophysiological predictor. The effective group exhibited a significantly longer EEG seizure duration compared to the ineffective group, with seizure duration strongly associated with treatment response (OR = 1.183, p < 0.001). These findings are consistent with prior research (43, 44) and suggest that monitoring EEG seizure duration provides useful information when titrating treatment.

No independent predictive value was found for the seizure index or suppression index, consistent with earlier studies (45, 46). This study employed a personalised parameter adjustment strategy, modifying stimulus charge, current, frequency, and duration to balance efficacy with tolerability. These clinician-led adjustments, although beneficial for individual outcomes, may obscure population-level dose–response relationships due to variability and limited sample size. This could explain why no significant associations were observed between stimulation parameters and clinical outcomes in our analysis. Future studies with larger cohorts are needed to elucidate the complex interplay between electrical dosing and neural plasticity.

### 4.4 Strengths and limitations

This study presents a comprehensive multidimensional evaluation, encompassing demographic, clinical, and electrophysiological factors, thereby enhancing the representativeness and practical relevance of the findings. The integration of diverse predictors strengthens the external validity and clinical applicability of the results. However, several limitations must be acknowledged:

First, the retrospective, observational design limits causal inference. Treatment assignment and parameter adjustments were determined by clinical teams rather than by protocolised allocation, so unmeasured confounding and indication bias may have influenced both treatment choices and outcomes despite multivariable adjustment. Second, the study was conducted at a single tertiary psychiatric hospital; local clinical practice, patient demographics and device-setting routines may differ from other centres, which restricts the generalisability of our findings. Third, we included only patients who completed the six-session MECT course with complete pre- and post-treatment PANSS data. This inclusion criterion risks survivorship (selection) bias: patients who discontinued early because of adverse events, clinical deterioration, practical reasons or early non-response were not represented in the primary analysis and may differ systematically from completers. Although we performed sensitivity analyses treating early dropouts as non-responders (reported in the **Supplementary Material**), prospective intention-to-treat data would more robustly estimate real-world effectiveness.Fourth, the study did not include formal cognitive assessments or longer-term follow-up. As a result, we cannot comment on the cognitive safety profile of the six-session regimen, nor on durability of response or relapse rates beyond the immediate post-treatment window. These outcomes are clinically important when weighing short-term symptom improvement against potential cognitive adverse effects and relapse prevention strategies. Fifth, although PANSS ratings were performed by two trained psychiatrists under a standardised protocol, we did not compute inter-rater reliability (e.g., ICC or kappa) for this dataset; future prospective work should include formal rater-training and reliability testing. Finally, several electrophysiological and device parameters (for example, seizure index, suppression index and impedance measures) are susceptible to measurement variability (influenced by electrode placement, muscle relaxation, amplifier settings and artefact) and were adjusted in real-time by clinicians; this pragmatic approach improves individual care but reduces the internal control required to precisely estimate dose–response relationships. Taken together, these limitations motivate prospective, multicentre, randomised or adaptive-design studies with standardised titration algorithms, comprehensive cognitive testing and longer follow-up to confirm and extend the present findings.

Future research should aim to address these limitations through multicentre, prospective, randomised controlled trials to validate the present findings. Additionally, exploring the associations between stimulation modalities (e.g., unilateral vs bilateral electrode placement, stimulation frequency variations) and both therapeutic outcomes and adverse effects will be essential. The integration of neuroimaging, electrophysiological indices, and biological markers may also enable the development of more personalised and precise MECT protocols.

## 5 Conclusion

MECT is an effective therapeutic strategy for patients with schizophrenia, particularly during the acute phase. Treatment efficacy is influenced by several key factors, including age, illness duration, first-episode status, baseline severity of positive symptoms, and EEG seizure duration. These variables should be considered when formulating individualised MECT treatment plans, with the aim of maximising clinical efficacy and informing optimised decision-making in psychiatric practice.

## Data Availability

The datasets presented in this article are not readily available because access requires approval from the Fourth People’s Hospital of Nantong City, Jiangsu Province, China. Requests to access the datasets should be directed to XW, xueting_wang2021@126.com.
