# Supplementary material for: Efficacy and influencing factors of modified electroconvulsive therapy for schizophrenia: a real-world retrospective observational study
Source: Front Psychiatry. 2025 Oct 3;16:1654151. doi: 10.3389/fpsyt.2025.1654151 (PMC12531135; doi:10.3389/fpsyt.2025.1654151)

**Supplementary materials 1**

**Seizure adequacy criteria and dose‑titration algorithm**

1. Seizure adequacy (EEG and motor criteria)

(1)Primary (EEG) criterion: a generalised, synchronous ictal EEG pattern followed by clear post‑ictal suppression. Seizure duration on the EEG trace of ≥20 seconds was considered adequate for clinical purposes. Post‑ictal suppression was visually inspected and used to judge seizure quality.

(2)Secondary (motor) criterion: observable tonic‑clonic activity in an isolated limb (tourniquet technique used to preserve a motor response despite muscle relaxant), with motor seizure duration ≥15 seconds counted as supporting evidence of an adequate seizure.

EEG seizure duration was treated as the principal marker of seizure quality for adjustment decisions because muscle relaxants blunt motor signs; when EEG and motor markers conflicted, EEG findings took precedence.

1. Dose‑titration algorithm

(1)Initial dose: The first session’s stimulus dose was set according to an age‑based approach consistent with widely used clinical practice (commonly referenced as the ‘half‑age’ method or manufacturer age‑based table), as described in the Methods. This approach uses patient age to guide an initial estimate of stimulus charge while remaining within device safety limits.

(2)Incremental escalation: If the initial stimulus produced an inadequate seizure (EEG duration <20 s or insufficient post‑ictal suppression), the stimulus charge was increased in small, clinician‑directed steps (typically 5–10% increases of delivered charge or the next pre‑set device increment) in subsequent sessions until an adequate seizure was achieved or the clinician judged further escalation unsafe.

(3)Post‑ictal suppression target: Clinicians aimed for robust post‑ictal suppression as an index of seizure quality. Where measurable, higher post‑ictal suppression was preferred (clinically targeted for the treating team but not required for inclusion). Post‑ictal suppression was considered alongside seizure duration when making adjustments.

(4)Procedural safety limits: All stimulus adjustments respected device and institutional safety limits. The maximum device settings were not exceeded, and decisions to escalate were balanced against adverse effects, patient tolerance and anaesthetic considerations.

(5)Other stimulation parameters: Pulse width, frequency, and train duration generally followed Thymatron® default settings; these parameters were only altered when clinically indicated (for example, to improve seizure quality or reduce side effects).

**Supplementary materials 2**

**Table 1. Average Olanzapine Equivalent Doses of Antipsychotic Medications Used in Patients with Schizophrenia**

| **Drug** | **Average Olanzapine equivalents** |
| --- | --- |
| Olanzapine | 1 |
| Quetiapine | 40 |
| Clozapine | 30 |
| Amisulpride | 40 |
| Risperidone | 0.5 |
| Aripiprazole | 1.5 |
| Acetophenazine | 5 |
| Ziprasidone | 8 |
| Fluphenazine | 1 |
| Paliperidone | 0.6 |
| Penfluridol | 0.6 |
| Haloperidol | 0.8 |
| Chlorpromazine | 30 |
| Perphenazine | 3 |

Note: Dose equivalents are based on previously established conversion formulas referenced in the study.

**Supplementary materials 3 Methods for VIF-based variable selection**

In the model analysis, all candidate variables—including demographic characteristics, health status, clinical features, MECT treatment parameters, and baseline PANSS scores—were entered into the initial model. The preliminary mean Variance Inflation Factor (VIF) was 3990.93, indicating severe multicollinearity. Based on clinical relevance and statistical considerations, five variables were subsequently excluded: static impedance (VIF = 70,873.78), stimulus charge (VIF = 51,036.96), stimulus frequency (VIF = 5,608.05), dynamic impedance (VIF = 1,824.90), and total PANSS score (VIF = 882.17). After exclusion, the mean VIF of the remaining 12 variables was reduced to 1.74, and these variables were retained for the final multivariate logistic regression analysis.

**Table 2. Variance Inflation Factor (VIF) Analysis of Predictor Variables**

| Variable | Full Model VIF | Reduced Model VIF |
| --- | --- | --- |
| Age group (years) |  |  |
| 30–40 | 2.01 | 2.00 |
| 40–50 | 2.08 | 2.04 |
| 50–60 | 1.85 | 1.68 |
| ≥60 | 1.83 | 1.73 |
| Sex (Female) | 2.16 | 2.09 |
| Education level |  |  |
| Upper secondary & vocational training | 1.28 | 1.25 |
| Tertiary | 1.26 | 1.25 |
| Marital status (Single) | 1.23 | 1.18 |
| Residence (Rural) | 1.3 | 1.22 |
| BMI | 1.52 | 1.46 |
| Family history of psychosis | 1.18 | 1.14 |
| Smoking (Yes) | 1.85 | 1.76 |
| Alcohol consumption |  |  |
| Moderate drinking | 1.24 | 1.23 |
| Harmful drinking | 1.58 | 1.48 |
| Antipsychotic dose (mg/day) | 1.17 | 1.12 |
| Duration of illness |  |  |
| 5–10 years | 7.48 | 1.94 |
| 10–15 years | 15.18 | 2.19 |
| ≥15 years | 57.76 | 5.58 |
| First episode (Yes) | 1.23 | 1.21 |
| MECT parameters |  |  |
| EEG seizure duration | 807.24 | 3.08 |
| Seizure Index | 1.19 | 1.17 |
| Suppression Index | 1.12 | 1.1 |
| Static impedance | 70873.78 | Excluded |
| Dynamic impedance | 1824.9 | Excluded |
| Energy percentage | 1.16 | 1.13 |
| Stimulus charge | 51036.96 | Excluded |
| Stimulus current | 1.13 | 1.11 |
| Stimulus frequency | 5608.05 | Excluded |
| Stimulus duration | 1.20 | 1.18 |
| Baseline PANSS subscale scores |  |  |
| Negative subscale | 106.31 | 1.45 |
| Positive subscale | 157.73 | 2.51 |
| General psychopathology subscale | 292.58 | 2.44 |
| Total score | 882.17 | Excluded |
| Mean VIF | 3990.93 | 1.74 |

**Supplementary materials 4 Sensitivity and robustness analysis**

1. Using alternative responder thresholds ≥30%

| **Variable** | **OR** | **SE** | **z** | **p-value** | **95% CI** |
| --- | --- | --- | --- | --- | --- |
| **Age Group** |  |  |  |  |  |
| <30 (Ref.) | – | – | – | – | – |
| 30–40 | 0.558 | 0.670 | -0.490 | 0.627 | 0.053 - 5.867 |
| 40–50 | 0.386 | 0.430 | -0.860 | 0.392 | 0.044 - 3.420 |
| 50–60 | 0.202 | 0.250 | -1.290 | 0.196 | 0.018 - 2.281 |
| ≥60 | 0.027 | 0.033 | -2.950 | 0.003 | 0.002 - 0.295 |
| **Sex** |  |  |  |  |  |
| Male (Ref.) | – | – | – | – | – |
| Female | 7.502 | 6.832 | 2.210 | 0.027 | 1.259 - 44.700 |
| **Education Level** |  |  |  |  |  |
| Less than lower secondary | – | – | – | – | – |
| upper secondary & vocational training | 0.295 | 0.308 | -1.170 | 0.242 | 0.038 - 2.276 |
| tertiary | 0.101 | 0.109 | -2.130 | 0.033 | 0.012 - 0.832 |
| **Marital Status** |  |  |  |  |  |
| With partner (Ref.) | – | – | – | – | – |
| Single | 0.268 | 0.255 | -1.380 | 0.167 | 0.042 - 1.731 |
| **Residence** |  |  |  |  |  |
| Urban (Ref.) | – | – | – | – | – |
| Rural | 0.139 | 0.107 | -2.570 | 0.010 | 0.031 - 0.627 |
| **BMI** | 1.017 | 0.047 | 0.350 | 0.723 | 0.928 - 1.114 |
| **Family History of SCZ** |  |  |  |  |  |
| No (Ref.) | – | – | – | – | – |
| Yes | 8.227 | 10.115 | 1.710 | 0.087 | 0.739 - 91.578 |
| **Smoking** |  |  |  |  |  |
| No (Ref.) | – | – | – | – | – |
| Yes | 1.393 | 1.096 | 0.420 | 0.674 | 0.298 - 6.510 |
| **Alcohol Use** |  |  |  |  |  |
| Non-drinker (Ref.) | – | – | – | – | – |
| Moderate drinking | 4.743 | 5.587 | 1.320 | 0.186 | 0.471 - 47.717 |
| Harmful drinking | 0.550 | 0.518 | -0.630 | 0.526 | 0.087 - 3.485 |
| **Antipsychotic Dose** | 0.980 | 0.047 | -0.410 | 0.678 | 0.893 - 1.076 |
| **Illness duration** |  |  |  |  |  |
| <5 years (Ref.) | – | – | – | – | – |
| 5–10 years | 0.183 | 0.262 | -1.190 | 0.235 | 0.011 - 3.008 |
| 10–15 years | 0.010 | 0.014 | -3.270 | 0.001 | 0.001 - 0.158 |
| ≥15 years | 0.028 | 0.040 | -2.500 | 0.013 | 0.002 - 0.462 |
| **First-episode status** |  |  |  |  |  |
| No (Ref.) | – | – | – | – | – |
| Yes | 2.848 | 2.247 | 1.330 | 0.185 | 0.607 - 13.371 |
| **MECT Parameters** |  |  |  |  |  |
| EEG Seizure Duration | 1.110 | 0.041 | 2.850 | 0.004 | 1.033 - 1.193 |
| ASEI | 0.692 | 0.912 | -0.280 | 0.780 | 0.052 - 9.154 |
| PSI | 1.014 | 0.036 | 0.380 | 0.703 | 0.945 - 1.088 |
| Energy Percentage | 1.023 | 0.025 | 0.940 | 0.347 | 0.975 - 1.073 |
| Stimulus Current | 4.722 | 15.047 | 0.490 | 0.626 | 0.009 - 2436.321 |
| Stimulus Duration | 0.281 | 0.145 | -2.460 | 0.014 | 0.102 - 0.773 |
| **Pre-treatment PANSS Scores** |  |  |  |  |  |
| Negative symptoms | 1.260 | 0.099 | 2.950 | 0.003 | 1.081 - 1.470 |
| Positive symptoms | 0.964 | 0.051 | -0.690 | 0.493 | 0.868 - 1.070 |
| General psychopathology | 1.137 | 0.050 | 2.930 | 0.003 | 1.044 - 1.239 |

1. An intention-to-treat (worst-case) analysis treating all dropped patients as non-responders followed by logistic regression.

| **Variable** | **OR** | **SE** | **z** | **p-value** | **95% CI** |
| --- | --- | --- | --- | --- | --- |
| **Age Group** |  |  |  |  |  |
| <30 (Ref.) | – | – | – | – | – |
| 30–40 | 1.209 | 0.875 | 0.260 | 0.793 | 0.293 - 4.991 |
| 40–50 | 1.057 | 0.801 | 0.070 | 0.942 | 0.239 - 4.669 |
| 50–60 | 0.111 | 0.095 | -2.560 | 0.010 | 0.021 - 0.597 |
| ≥60 | 0.078 | 0.065 | -3.050 | 0.002 | 0.015 - 0.402 |
| **Sex** |  |  |  |  |  |
| Male (Ref.) | – | – | – | – | – |
| Female | 2.763 | 1.824 | 1.540 | 0.124 | 0.757 - 10.078 |
| **Education Level** |  |  |  |  |  |
| Less than lower secondary | – | – | – | – | – |
| upper secondary & vocational training | 0.993 | 0.832 | -0.010 | 0.993 | 0.192 - 5.129 |
| tertiary | 0.527 | 0.462 | -0.730 | 0.465 | 0.095 - 2.937 |
| **Marital Status** |  |  |  |  |  |
| With partner (Ref.) | – | – | – | – | – |
| Single | 0.725 | 0.538 | -0.430 | 0.665 | 0.169 - 3.109 |
| **Residence** |  |  |  |  |  |
| Urban (Ref.) | – | – | – | – | – |
| Rural | 0.579 | 0.300 | -1.050 | 0.292 | 0.209 - 1.599 |
| **BMI** | 1.059 | 0.056 | 1.090 | 0.274 | 0.955 - 1.175 |
| **Family History of SCZ** |  |  |  |  |  |
| No (Ref.) | – | – | – | – | – |
| Yes | 3.113 | 2.556 | 1.380 | 0.166 | 0.623 - 15.557 |
| **Smoking** |  |  |  |  |  |
| No (Ref.) | – | – | – | – | – |
| Yes | 1.793 | 1.221 | 0.860 | 0.391 | 0.472 - 6.814 |
| **Alcohol Use** |  |  |  |  |  |
| Non-drinker (Ref.) | – | – | – | – | – |
| Moderate drinking | 3.689 | 3.097 | 1.560 | 0.120 | 0.712 - 19.116 |
| Harmful drinking | 1.808 | 1.304 | 0.820 | 0.411 | 0.440 - 7.429 |
| **Antipsychotic Dose** | 0.983 | 0.035 | -0.500 | 0.619 | 0.917 - 1.053 |
| **Illness duration** |  |  |  |  |  |
| <5 years (Ref.) | – | – | – | – | – |
| 5–10 years | 0.193 | 0.205 | -1.550 | 0.122 | 0.024 - 1.550 |
| 10–15 years | 0.028 | 0.036 | -2.840 | 0.004 | 0.002 - 0.331 |
| ≥15 years | 0.003 | 0.004 | -4.120 | 0.000 | 0.000 - 0.047 |
| **First-episode status** |  |  |  |  |  |
| No (Ref.) | – | – | – | – | – |
| Yes | 6.537 | 4.060 | 3.020 | 0.003 | 1.935 - 22.083 |
| **MECT Parameters** |  |  |  |  |  |
| EEG Seizure Duration | 1.183 | 0.044 | 4.550 | 0.000 | 1.100 - 1.272 |
| ASEI | 0.202 | 0.226 | -1.430 | 0.153 | 0.023 - 1.809 |
| PSI | 0.963 | 0.026 | -1.400 | 0.162 | 0.914 - 1.015 |
| Energy Percentage | 1.005 | 0.020 | 0.270 | 0.787 | 0.966 - 1.046 |
| Stimulus Current | 0.210 | 0.470 | -0.700 | 0.486 | 0.003 - 17.005 |
| Stimulus Duration | 0.925 | 0.352 | -0.210 | 0.837 | 0.439 - 1.949 |
| **Pre-treatment PANSS Scores** |  |  |  |  |  |
| Negative symptoms | 1.325 | 0.071 | 5.240 | 0.000 | 1.193 - 1.472 |
| Positive symptoms | 0.990 | 0.051 | -0.190 | 0.848 | 0.896 - 1.094 |
| General psychopathology | 0.960 | 0.027 | -1.440 | 0.150 | 0.908 - 1.015 |

1. Ordinal logistic model using the four pre-specified categories (≥75%, 50–74%, 25–49%, <25%)

| **Variable** | **OR** | **SE** | **z** | **p-value** | **95% CI** |
| --- | --- | --- | --- | --- | --- |
| **Age Group** |  |  |  |  |  |
| <30 (Ref.) | – | – | – | – | – |
| 30–40 | 1.143 | 0.756 | 0.200 | 0.840 | 0.313 - 4.180 |
| 40–50 | 0.786 | 0.507 | -0.370 | 0.709 | 0.221 - 2.786 |
| 50–60 | 0.216 | 0.147 | -2.250 | 0.024 | 0.057 - 0.819 |
| ≥60 | 0.140 | 0.093 | -2.940 | 0.003 | 0.038 - 0.518 |
| **Sex** |  |  |  |  |  |
| Male (Ref.) | – | – | – | – | – |
| Female | 2.938 | 1.582 | 2.000 | 0.045 | 1.023 - 8.441 |
| **Education Level** |  |  |  |  |  |
| Less than lower secondary | – | – | – | – | – |
| upper secondary & vocational training | 0.777 | 0.493 | -0.400 | 0.691 | 0.224 - 2.696 |
| tertiary | 0.347 | 0.257 | -1.430 | 0.153 | 0.081 - 1.482 |
| **Marital Status** |  |  |  |  |  |
| With partner (Ref.) | – | – | – | – | – |
| Single | 0.603 | 0.380 | -0.800 | 0.422 | 0.175 - 2.074 |
| **Residence** |  |  |  |  |  |
| Urban (Ref.) | – | – | – | – | – |
| Rural | 0.638 | 0.263 | -1.090 | 0.275 | 0.284 - 1.431 |
| **BMI** | 1.037 | 0.036 | 1.030 | 0.303 | 0.968 - 1.110 |
| **Family History of SCZ** |  |  |  |  |  |
| No (Ref.) | – | – | – | – | – |
| Yes | 2.314 | 1.507 | 1.290 | 0.198 | 0.646 - 8.292 |
| **Smoking** |  |  |  |  |  |
| No (Ref.) | – | – | – | – | – |
| Yes | 1.491 | 0.803 | 0.740 | 0.459 | 0.518 - 4.286 |
| **Alcohol Use** |  |  |  |  |  |
| Non-drinker (Ref.) | – | – | – | – | – |
| Moderate drinking | 2.500 | 1.635 | 1.400 | 0.161 | 0.694 - 9.005 |
| Harmful drinking | 1.310 | 0.783 | 0.450 | 0.652 | 0.406 - 4.229 |
| **Antipsychotic Dose** | 0.973 | 0.029 | -0.930 | 0.351 | 0.918 - 1.031 |
| **Illness duration** |  |  |  |  |  |
| <5 years (Ref.) | – | – | – | – | – |
| 5–10 years | 0.258 | 0.225 | -1.560 | 0.120 | 0.047 - 1.423 |
| 10–15 years | 0.089 | 0.084 | -2.560 | 0.010 | 0.014 - 0.568 |
| ≥15 years | 0.048 | 0.044 | -3.310 | 0.001 | 0.008 - 0.289 |
| **First-episode status** |  |  |  |  |  |
| No (Ref.) | – | – | – | – | – |
| Yes | 4.850 | 2.310 | 3.320 | 0.001 | 1.907 - 12.335 |
| **MECT Parameters** |  |  |  |  |  |
| EEG Seizure Duration | 1.099 | 0.025 | 4.080 | 0.000 | 1.050 - 1.150 |
| ASEI | 0.160 | 0.143 | -2.060 | 0.040 | 0.028 - 0.918 |
| PSI | 0.988 | 0.021 | -0.590 | 0.556 | 0.948 - 1.029 |
| Energy Percentage | 0.987 | 0.016 | -0.860 | 0.391 | 0.956 - 1.018 |
| Stimulus Current | 0.994 | 1.815 | 0.000 | 0.998 | 0.028 - 35.609 |
| Stimulus Duration | 0.828 | 0.253 | -0.620 | 0.538 | 0.455 - 1.508 |
| **Pre-treatment PANSS Scores** |  |  |  |  |  |
| Negative symptoms | 1.248 | 0.052 | 5.340 | 0.000 | 1.151 - 1.354 |
| Positive symptoms | 1.010 | 0.039 | 0.270 | 0.788 | 0.937 - 1.089 |
| General psychopathology | 1.000 | 0.024 | 0.010 | 0.995 | 0.954 - 1.049 |

1. Using multivariable linear regression using the same candidate predictors.

R² = 0.434；Adjusted R² = 0.358；RMSE = 0.104

| **Variable** | **OR** | **SE** | **z** | **p-value** | **95% CI** |
| --- | --- | --- | --- | --- | --- |
| **Age Group** |  |  |  |  |  |
| <30 (Ref.) | – | – | – | – | – |
| 30–40 | -0.013 | 0.022 | -0.580 | 0.564 | -0.055 - 0.030 |
| 40–50 | -0.018 | 0.022 | -0.830 | 0.407 | -0.061 - 0.025 |
| 50–60 | -0.047 | 0.026 | -1.820 | 0.070 | -0.098 - 0.004 |
| ≥60 | -0.098 | 0.027 | -3.640 | 0.000 | -0.151 - -0.045 |
| **Sex** |  |  |  |  |  |
| Male (Ref.) | – | – | – | – | – |
| Female | 0.034 | 0.020 | 1.720 | 0.087 | -0.005 - 0.073 |
| **Education Level** |  |  |  |  |  |
| Less than lower secondary | – | – | – | – | – |
| upper secondary & vocational training | -0.008 | 0.022 | -0.350 | 0.724 | -0.050 - 0.035 |
| tertiary | -0.038 | 0.028 | -1.350 | 0.178 | -0.093 - 0.017 |
| **Marital Status** |  |  |  |  |  |
| With partner (Ref.) | – | – | – | – | – |
| Single | -0.005 | 0.022 | -0.210 | 0.837 | -0.048 - 0.039 |
| **Residence** |  |  |  |  |  |
| Urban (Ref.) | – | – | – | – | – |
| Rural | -0.034 | 0.015 | -2.190 | 0.030 | -0.065 - -0.003 |
| **BMI** | 0.001 | 0.002 | 0.450 | 0.656 | -0.003 - 0.004 |
| **Family History of SCZ** |  |  |  |  |  |
| No (Ref.) | – | – | – | – | – |
| Yes | 0.025 | 0.021 | 1.220 | 0.225 | -0.015 - 0.065 |
| **Smoking** |  |  |  |  |  |
| No (Ref.) | – | – | – | – | – |
| Yes | 0.018 | 0.019 | 0.940 | 0.347 | -0.020 - 0.056 |
| **Alcohol Use** |  |  |  |  |  |
| Non-drinker (Ref.) | – | – | – | – | – |
| Moderate drinking | 0.027 | 0.025 | 1.080 | 0.283 | -0.022 - 0.075 |
| Harmful drinking | -0.004 | 0.020 | -0.190 | 0.853 | -0.044 - 0.037 |
| **Antipsychotic Dose** | 0.000 | 0.001 | -0.180 | 0.857 | -0.002 - 0.002 |
| **Illness duration** |  |  |  |  |  |
| <5 years (Ref.) | – | – | – | – | – |
| 5–10 years | -0.060 | 0.033 | -1.780 | 0.076 | -0.125 - 0.006 |
| 10–15 years | -0.114 | 0.035 | -3.230 | 0.001 | -0.184 - -0.044 |
| ≥15 years | -0.129 | 0.036 | -3.570 | 0.000 | -0.200 - -0.058 |
| **First-episode status** |  |  |  |  |  |
| No (Ref.) | – | – | – | – | – |
| Yes | 0.040 | 0.016 | 2.540 | 0.012 | 0.009 - 0.070 |
| **MECT Parameters** |  |  |  |  |  |
| EEG Seizure Duration | 0.004 | 0.001 | 4.400 | 0.000 | 0.002 - 0.005 |
| ASEI | -0.048 | 0.033 | -1.430 | 0.155 | -0.114 - 0.018 |
| PSI | 0.000 | 0.001 | 0.200 | 0.840 | -0.001 - 0.002 |
| Energy Percentage | 0.000 | 0.001 | -0.090 | 0.925 | -0.001 - 0.001 |
| Stimulus Current | 0.064 | 0.070 | 0.900 | 0.367 | -0.075 - 0.202 |
| Stimulus Duration | -0.021 | 0.012 | -1.770 | 0.078 | -0.044 - 0.002 |
| **Pre-treatment PANSS Scores** |  |  |  |  |  |
| Negative symptoms | 0.006 | 0.001 | 4.150 | 0.000 | 0.003 - 0.008 |
| Positive symptoms | 0.001 | 0.002 | 0.740 | 0.459 | -0.002 - 0.004 |
| General psychopathology | 0.002 | 0.001 | 2.130 | 0.035 | 0.000 - 0.004 |

1. Using beta regression to test robustness.

| **Variable** | **OR** | **SE** | **z** | **p-value** | **95% CI** |
| --- | --- | --- | --- | --- | --- |
| **Age Group** |  |  |  |  |  |
| <30 (Ref.) | – | – | – | – | – |
| 30–40 | -0.080 | 0.095 | -0.850 | 0.398 | -0.265 - 0.105 |
| 40–50 | -0.092 | 0.094 | -0.970 | 0.330 | -0.277 - 0.093 |
| 50–60 | -0.194 | 0.113 | -1.710 | 0.087 | -0.415 - 0.028 |
| ≥60 | -0.411 | 0.119 | -3.460 | 0.001 | -0.644 - -0.178 |
| **Sex** |  |  |  |  |  |
| Male (Ref.) | – | – | – | – | – |
| Female | 0.163 | 0.086 | 1.890 | 0.059 | -0.006 - 0.332 |
| **Education Level** |  |  |  |  |  |
| Less than lower secondary | – | – | – | – | – |
| upper secondary & vocational training | -0.064 | 0.094 | -0.680 | 0.496 | -0.249 - 0.120 |
| tertiary | -0.198 | 0.123 | -1.610 | 0.108 | -0.440 - 0.044 |
| **Marital Status** |  |  |  |  |  |
| With partner (Ref.) | – | – | – | – | – |
| Single | -0.023 | 0.096 | -0.240 | 0.809 | -0.211 - 0.165 |
| **Residence** |  |  |  |  |  |
| Urban (Ref.) | – | – | – | – | – |
| Rural | -0.148 | 0.068 | -2.190 | 0.028 | -0.280 - -0.016 |
| **BMI** | -0.001 | 0.007 | -0.080 | 0.936 | -0.015 - 0.014 |
| **Family History of SCZ** |  |  |  |  |  |
| No (Ref.) | – | – | – | – | – |
| Yes | 0.129 | 0.090 | 1.430 | 0.151 | -0.047 - 0.305 |
| **Smoking** |  |  |  |  |  |
| No (Ref.) | – | – | – | – | – |
| Yes | 0.080 | 0.084 | 0.950 | 0.342 | -0.085 - 0.246 |
| **Alcohol Use** |  |  |  |  |  |
| Non-drinker (Ref.) | – | – | – | – | – |
| Moderate drinking | 0.118 | 0.109 | 1.080 | 0.279 | -0.095 - 0.330 |
| Harmful drinking | -0.005 | 0.089 | -0.060 | 0.951 | -0.180 - 0.169 |
| **Antipsychotic Dose** | -0.001 | 0.005 | -0.130 | 0.896 | -0.010 - 0.009 |
| **Illness duration** |  |  |  |  |  |
| <5 years (Ref.) | – | – | – | – | – |
| 5–10 years | -0.276 | 0.145 | -1.900 | 0.058 | -0.561 - 0.009 |
| 10–15 years | -0.489 | 0.153 | -3.190 | 0.001 | -0.789 - -0.189 |
| ≥15 years | -0.551 | 0.158 | -3.480 | 0.000 | -0.861 - -0.241 |
| **First-episode status** |  |  |  |  |  |
| No (Ref.) | – | – | – | – | – |
| Yes | 0.167 | 0.068 | 2.470 | 0.014 | 0.034 - 0.300 |
| **MECT Parameters** |  |  |  |  |  |
| EEG Seizure Duration | 0.015 | 0.004 | 4.200 | 0.000 | 0.008 - 0.023 |
| ASEI | -0.237 | 0.145 | -1.630 | 0.104 | -0.522 - 0.048 |
| PSI | 0.001 | 0.003 | 0.370 | 0.710 | -0.005 - 0.008 |
| Energy Percentage | -0.001 | 0.003 | -0.220 | 0.828 | -0.006 - 0.005 |
| Stimulus Current | 0.289 | 0.308 | 0.940 | 0.347 | -0.314 - 0.893 |
| Stimulus Duration | -0.067 | 0.051 | -1.320 | 0.187 | -0.167 - 0.033 |
| **Pre-treatment PANSS Scores** |  |  |  |  |  |
| Negative symptoms | 0.024 | 0.006 | 4.010 | 0.000 | 0.012 - 0.036 |
| Positive symptoms | 0.010 | 0.007 | 1.470 | 0.141 | -0.003 - 0.023 |
| General psychopathology | 0.013 | 0.005 | 2.770 | 0.006 | 0.004 - 0.022 |

1. **Using EEG seisure during Per10s and PANNS positive Per 5 point**

| **Variable** | **OR** | **SE** | **z** | **p-value** | **95% CI** |
| --- | --- | --- | --- | --- | --- |
| **Age Group** |  |  |  |  |  |
| <30 (Ref.) | – | – | – | – | – |
| 30–40 | 1.209 | 0.875 | 0.260 | 0.793 | 0.293 - 4.991 |
| 40–50 | 1.057 | 0.801 | 0.070 | 0.942 | 0.239 - 4.669 |
| 50–60 | 0.111 | 0.095 | -2.560 | 0.010 | 0.021 - 0.597 |
| ≥60 | 0.078 | 0.065 | -3.050 | 0.002 | 0.015 - 0.402 |
| **Sex** |  |  |  |  |  |
| Male (Ref.) | – | – | – | – | – |
| Female | 2.763 | 1.824 | 1.540 | 0.124 | 0.757 - 10.078 |
| **Education Level** |  |  |  |  |  |
| Less than lower secondary | – | – | – | – | – |
| upper secondary & vocational training | 0.993 | 0.832 | -0.010 | 0.993 | 0.192 - 5.129 |
| tertiary | 0.527 | 0.462 | -0.730 | 0.465 | 0.095 - 2.937 |
| **Marital Status** |  |  |  |  |  |
| With partner (Ref.) | – | – | – | – | – |
| Single | 0.725 | 0.538 | -0.430 | 0.665 | 0.169 - 3.109 |
| **Residence** |  |  |  |  |  |
| Urban (Ref.) | – | – | – | – | – |
| Rural | 0.579 | 0.300 | -1.050 | 0.292 | 0.209 - 1.599 |
| **BMI** | 1.059 | 0.056 | 1.090 | 0.274 | 0.955 - 1.175 |
| **Family History of SCZ** |  |  |  |  |  |
| No (Ref.) | – | – | – | – | – |
| Yes | 3.113 | 2.556 | 1.380 | 0.166 | 0.623 - 15.557 |
| **Smoking** |  |  |  |  |  |
| No (Ref.) | – | – | – | – | – |
| Yes | 1.793 | 1.221 | 0.860 | 0.391 | 0.472 - 6.814 |
| **Alcohol Use** |  |  |  |  |  |
| Non-drinker (Ref.) | – | – | – | – | – |
| Moderate drinking | 3.689 | 3.097 | 1.560 | 0.120 | 0.712 - 19.116 |
| Harmful drinking | 1.808 | 1.304 | 0.820 | 0.411 | 0.440 - 7.429 |
| **Antipsychotic Dose** | 0.983 | 0.035 | -0.500 | 0.619 | 0.917 - 1.053 |
| **Illness duration** |  |  |  |  |  |
| <5 years (Ref.) | – | – | – | – | – |
| 5–10 years | 0.193 | 0.205 | -1.550 | 0.122 | 0.024 - 1.550 |
| 10–15 years | 0.028 | 0.036 | -2.840 | 0.004 | 0.002 - 0.331 |
| ≥15 years | 0.003 | 0.004 | -4.120 | 0.000 | 0.000 - 0.047 |
| **First-episode status** |  |  |  |  |  |
| No (Ref.) | – | – | – | – | – |
| Yes | 6.537 | 4.060 | 3.020 | 0.003 | 1.935 - 22.083 |
| **MECT Parameters** |  |  |  |  |  |
| EEG Seizure Duration | 5.375 | 1.989 | 4.550 | 0.000 | 2.603 - 11.100 |
| ASEI | 0.202 | 0.226 | -1.430 | 0.153 | 0.023 - 1.809 |
| PSI | 0.963 | 0.026 | -1.400 | 0.162 | 0.914 - 1.015 |
| Energy Percentage | 1.005 | 0.020 | 0.270 | 0.787 | 0.966 - 1.046 |
| Stimulus Current | 0.210 | 0.470 | -0.700 | 0.486 | 0.003 - 17.005 |
| Stimulus Duration | 0.925 | 0.352 | -0.210 | 0.837 | 0.439 - 1.949 |
| **Pre-treatment PANSS Scores** |  |  |  |  |  |
| Negative symptoms | 4.087 | 1.097 | 5.240 | 0.000 | 2.415 - 6.916 |
| Positive symptoms | 0.990 | 0.051 | -0.190 | 0.848 | 0.896 - 1.094 |
| General psychopathology | 0.960 | 0.027 | -1.440 | 0.150 | 0.908 - 1.015 |

1. **Interaction between first-episode status and illness duration**

| **Variable** | **OR** | **SE** | **z** | **p-value** | **95% CI** |
| --- | --- | --- | --- | --- | --- |
| **Age Group** |  |  |  |  |  |
| <30 (Ref.) | – | – | – | – | – |
| 30–40 | 0.206 | 0.731 | 0.280 | 0.778 | -1.226 - 1.638 |
| 40–50 | 0.060 | 0.759 | 0.080 | 0.936 | -1.427 - 1.548 |
| 50–60 | -2.201 | 0.859 | -2.560 | 0.010 | -3.884 - -0.518 |
| ≥60 | -2.544 | 0.840 | -3.030 | 0.002 | -4.190 - -0.897 |
| **Sex** |  |  |  |  |  |
| Male (Ref.) | – | – | – | – | – |
| Female | 1.031 | 0.667 | 1.550 | 0.122 | -0.275 - 2.337 |
| **Education Level** |  |  |  |  |  |
| Less than lower secondary | – | – | – | – | – |
| upper secondary & vocational training | -0.006 | 0.838 | -0.010 | 0.994 | -1.648 - 1.636 |
| tertiary | -0.645 | 0.879 | -0.730 | 0.462 | -2.367 - 1.076 |
| **Marital Status** |  |  |  |  |  |
| With partner (Ref.) | – | – | – | – | – |
| Single | -0.327 | 0.743 | -0.440 | 0.660 | -1.783 - 1.129 |
| **Residence** |  |  |  |  |  |
| Urban (Ref.) | – | – | – | – | – |
| Rural | -0.540 | 0.521 | -1.040 | 0.300 | -1.562 - 0.481 |
| **BMI** | 0.057 | 0.053 | 1.080 | 0.282 | -0.047 - 0.161 |
| **Family History of SCZ** |  |  |  |  |  |
| No (Ref.) | – | – | – | – | – |
| Yes | 1.132 | 0.820 | 1.380 | 0.167 | -0.475 - 2.740 |
| **Smoking** |  |  |  |  |  |
| No (Ref.) | – | – | – | – | – |
| Yes | 0.586 | 0.680 | 0.860 | 0.389 | -0.748 - 1.919 |
| **Alcohol Use** |  |  |  |  |  |
| Non-drinker (Ref.) | – | – | – | – | – |
| Moderate drinking | 1.307 | 0.840 | 1.560 | 0.120 | -0.340 - 2.954 |
| Harmful drinking | 0.597 | 0.721 | 0.830 | 0.408 | -0.816 - 2.009 |
| **Antipsychotic Dose** | -0.018 | 0.036 | -0.510 | 0.607 | -0.089 - 0.052 |
| **Illness duration** |  |  |  |  |  |
| <5 years (Ref.) | – | – | – | – | – |
| 5–10 years | -1.756 | 1.297 | -1.350 | 0.176 | -4.298 - 0.786 |
| 10–15 years | -3.764 | 1.849 | -2.040 | 0.042 | -7.388 - -0.141 |
| ≥15 years | -6.148 | 2.587 | -2.380 | 0.017 | -11.219 - -1.077 |
| **First-episode status** |  |  |  |  |  |
| No (Ref.) | – | – | – | – | – |
| Yes | 1.693 | 1.364 | 1.240 | 0.214 | -0.980 - 4.366 |
| **Illness duration # First-episode status** | 0.080 | 0.527 | 0.150 | 0.880 | -0.954-1.113 |
| **MECT Parameters** |  |  |  |  |  |
| EEG Seizure Duration | 0.169 | 0.037 | 4.530 | 0.000 | 0.096 - 0.242 |
| ASEI | -1.604 | 1.121 | -1.430 | 0.153 | -3.801 - 0.594 |
| PSI | -0.038 | 0.027 | -1.400 | 0.162 | -0.090 - 0.015 |
| Energy Percentage | 0.005 | 0.020 | 0.240 | 0.808 | -0.035 - 0.045 |
| Stimulus Current | -1.531 | 2.250 | -0.680 | 0.496 | -5.941 - 2.879 |
| Stimulus Duration | -0.067 | 0.386 | -0.170 | 0.862 | -0.825 - 0.690 |
| **Pre-treatment PANSS Scores** |  |  |  |  |  |
| Negative symptoms | 0.282 | 0.054 | 5.240 | 0.000 | 0.176 - 0.387 |
| Positive symptoms | -0.008 | 0.052 | -0.160 | 0.871 | -0.110 - 0.093 |
| General psychopathology | -0.041 | 0.029 | -1.420 | 0.155 | -0.097 - 0.015 |

1. **The exclusion of patients >60 year in predicting treatment response**

| **Variable** | **OR** | **SE** | **z** | **p-value** | **95% CI** |
| --- | --- | --- | --- | --- | --- |
| **Age Group** |  |  |  |  |  |
| <30 (Ref.) | – | – | – | – | – |
|  |  |  |  |  |  |
| 30–40 | 0.257 | 0.757 | 0.340 | 0.735 | -1.228 - 1.741 |
| 40–50 | 0.098 | 0.815 | 0.120 | 0.905 | -1.500 - 1.695 |
| 50–60 | -2.207 | 0.909 | -2.430 | 0.015 | -3.988 - -0.425 |
| **Sex** |  |  |  |  |  |
| Male (Ref.) | – | – | – | – | – |
| Female | 1.266 | 0.738 | 1.710 | 0.086 | -0.181 - 2.712 |
| **Education Level** |  |  |  |  |  |
| Less than lower secondary | – | – | – | – | – |
| upper secondary & vocational training | -0.618 | 0.921 | -0.670 | 0.503 | -2.423 - 1.188 |
| tertiary | -1.167 | 1.135 | -1.030 | 0.304 | -3.392 - 1.058 |
| **Marital Status** |  |  |  |  |  |
| With partner (Ref.) | – | – | – | – | – |
| Single | 0.000 | 0.000 | 0.000 | 0.000 | 0.000 - 0.000 |
| **Residence** |  |  |  |  |  |
| Urban (Ref.) | – | – | – | – | – |
| Rural | 0.059 | 0.069 | 0.860 | 0.391 | -0.076 - 0.194 |
| **BMI** | 0.000 | 0.000 | 0.000 | 0.000 | 0.000 - 0.000 |
| **Family History of SCZ** |  |  |  |  |  |
| No (Ref.) | – | – | – | – | – |
| Yes | 1.225 | 0.985 | 1.240 | 0.214 | -0.706 - 3.157 |
| **Smoking** |  |  |  |  |  |
| No (Ref.) | – | – | – | – | – |
| Yes | 0.562 | 0.786 | 0.710 | 0.475 | -0.978 - 2.102 |
| **Alcohol Use** |  |  |  |  |  |
| Non-drinker (Ref.) | – | – | – | – | – |
| Moderate drinking | 1.938 | 1.022 | 1.900 | 0.058 | -0.064 - 3.941 |
| Harmful drinking | 1.005 | 0.867 | 1.160 | 0.246 | -0.694 - 2.705 |
| **Antipsychotic Dose** | -0.028 | 0.041 | -0.670 | 0.505 | -0.109 - 0.053 |
| **Illness duration** |  |  |  |  |  |
| <5 years (Ref.) | – | – | – | – | – |
| 5–10 years | -1.873 | 1.282 | -1.460 | 0.144 | -4.387 - 0.640 |
| 10–15 years | -3.394 | 1.411 | -2.400 | 0.016 | -6.160 - -0.627 |
| ≥15 years | -6.139 | 1.680 | -3.650 | 0.000 | -9.432 - -2.847 |
| **First-episode status** |  |  |  |  |  |
| No (Ref.) | – | – | – | – | – |
| Yes | 1.790 | 0.665 | 2.690 | 0.007 | 0.488 - 3.093 |
| **MECT Parameters** |  |  |  |  |  |
| EEG Seizure Duration | 0.190 | 0.045 | 4.210 | 0.000 | 0.101 - 0.278 |
| ASEI | -2.211 | 1.344 | -1.640 | 0.100 | -4.845 - 0.424 |
| PSI | -0.049 | 0.030 | -1.610 | 0.108 | -0.108 - 0.011 |
| Energy Percentage | -0.005 | 0.025 | -0.220 | 0.830 | -0.054 - 0.043 |
| Stimulus Current | -0.179 | 3.046 | -0.060 | 0.953 | -6.150 - 5.791 |
| Stimulus Duration | 0.142 | 0.435 | 0.330 | 0.744 | -0.711 - 0.995 |
| **Pre-treatment PANSS Scores** |  |  |  |  |  |
| Negative symptoms | 0.303 | 0.061 | 4.950 | 0.000 | 0.183 - 0.423 |
| Positive symptoms | -0.001 | 0.069 | -0.020 | 0.985 | -0.137 - 0.134 |
| General psychopathology | -0.053 | 0.033 | -1.610 | 0.107 | -0.118 - 0.011 |

**Supplementary materials 5 Adverse Events**

|  | **Total** | **Ineffective Group** | **Effective Group** | ***x^2^*-value** | ***p*-value** |
| --- | --- | --- | --- | --- | --- |
| **N** | 237 | 70 | 167 |  |  |
| **AE[n(%)]** | 68  (28.7%) | 19  (27.1%) | 49  (29.3%) | 0.360 | 0.548 |
| headache | 40  (16.9%) | 13  (18.6%) | 27  (16.2%) | 1.7714 | 0.183 |
| transient confusion/delirium | 18  (7.6%) | 7  (10.0%) | 11  (6.6%) | 2.3964 | 0.122 |
| transient memory complaints | 12  (5.1%) | 5  (7.1%) | 7  (4.2%) | 2.2661 | 0.132 |

**Supplementary materials 6 The linearity of each continuous predictor in the logit using LOESS visualisation**


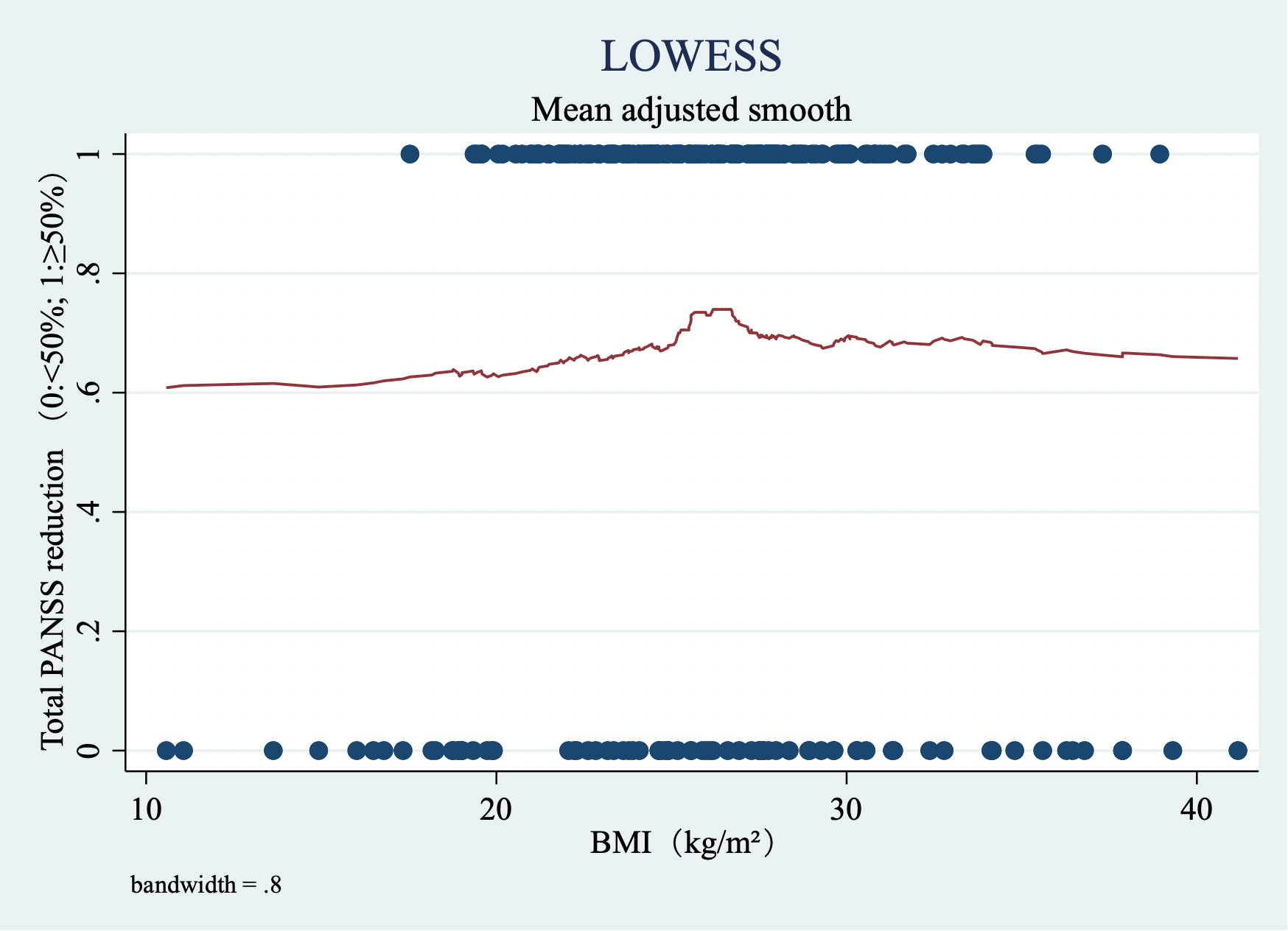

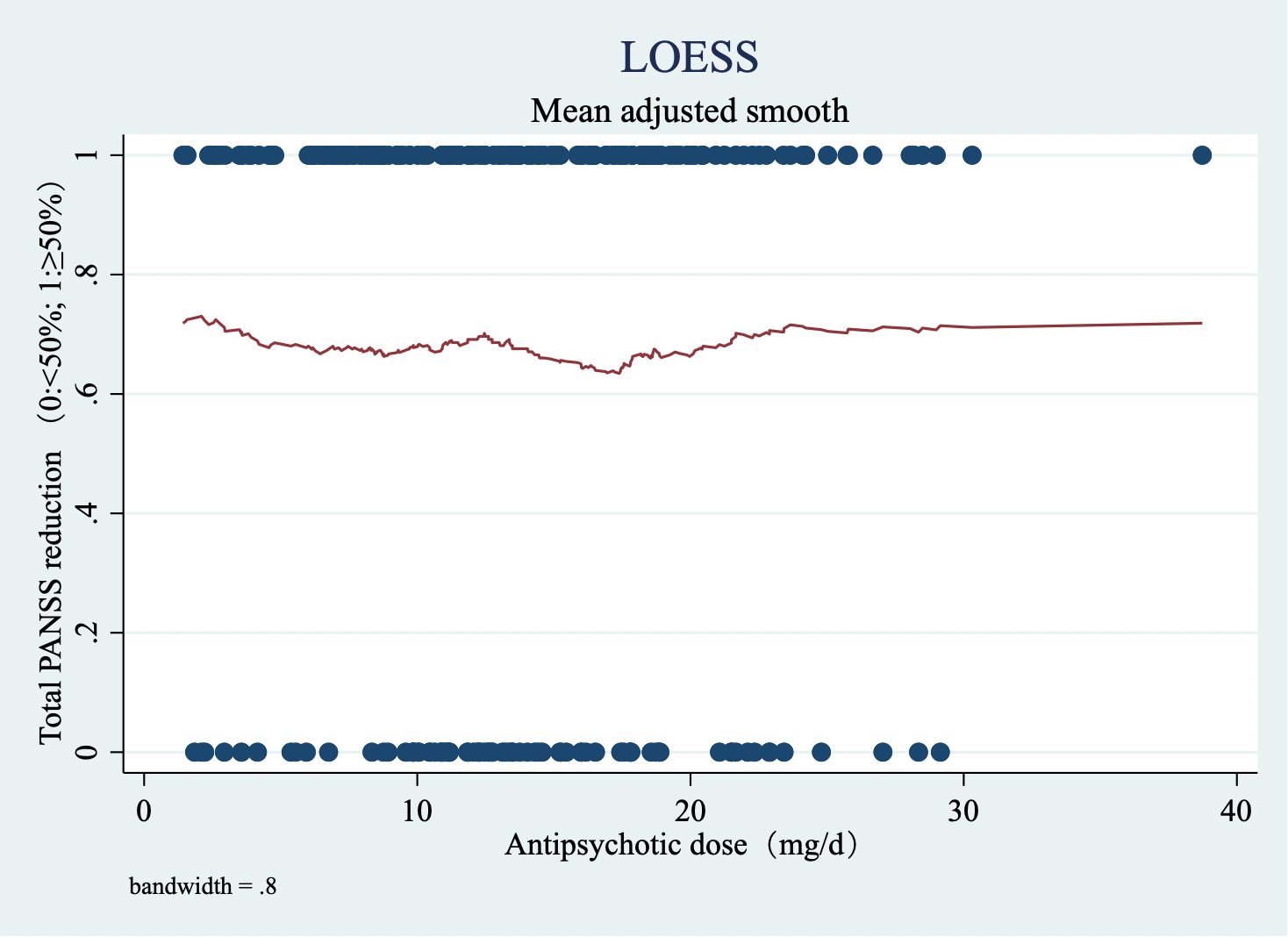


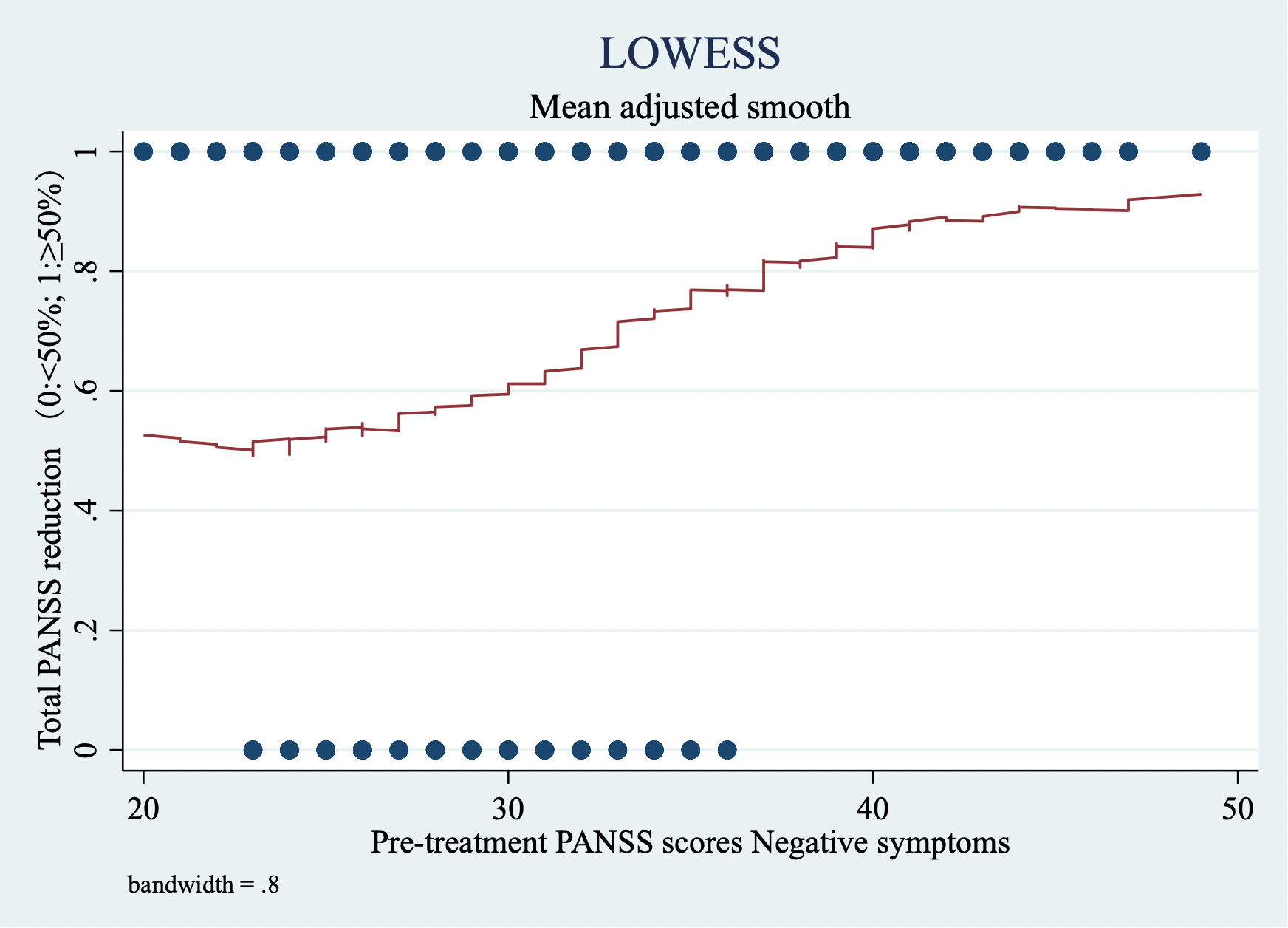

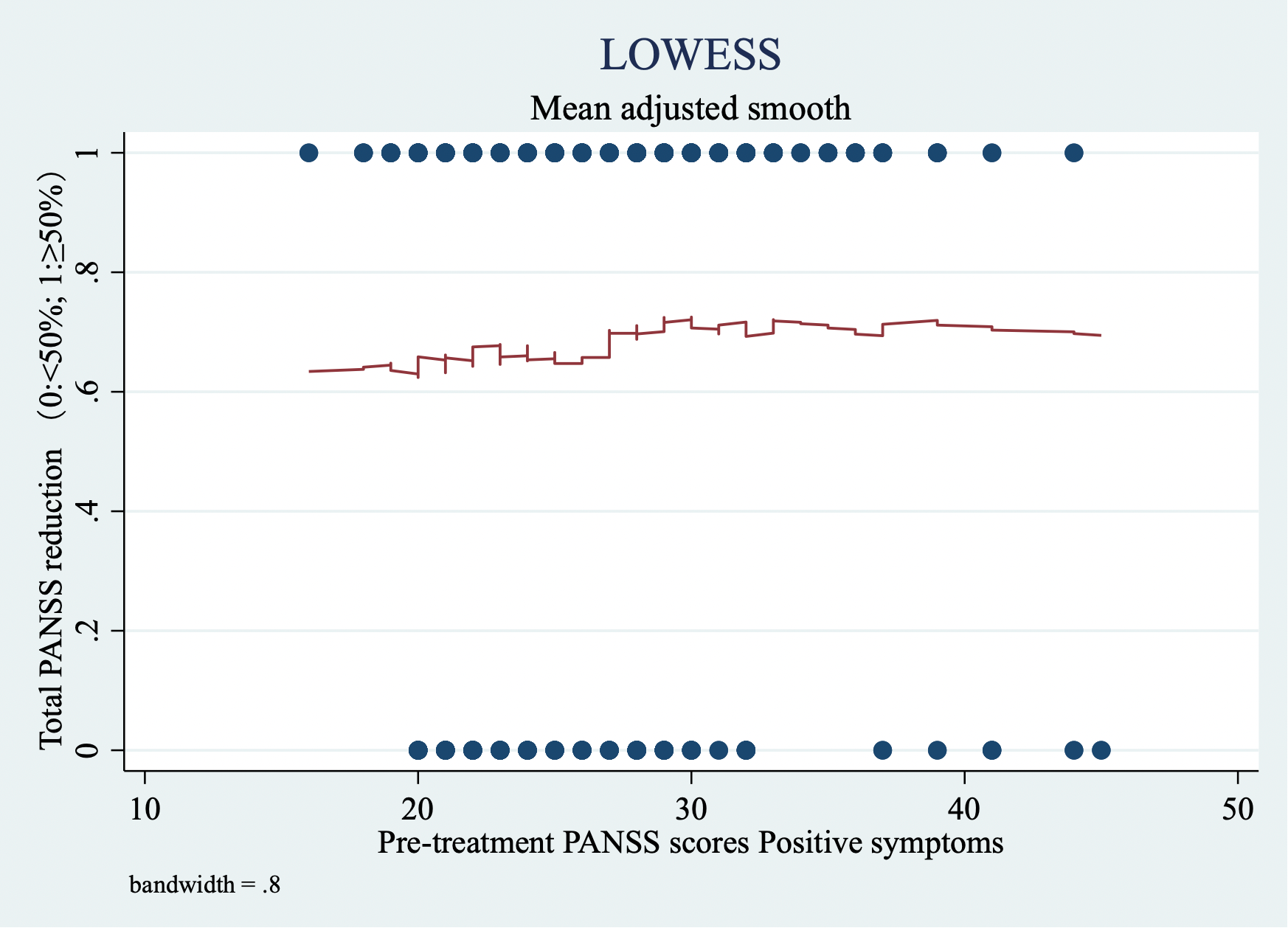


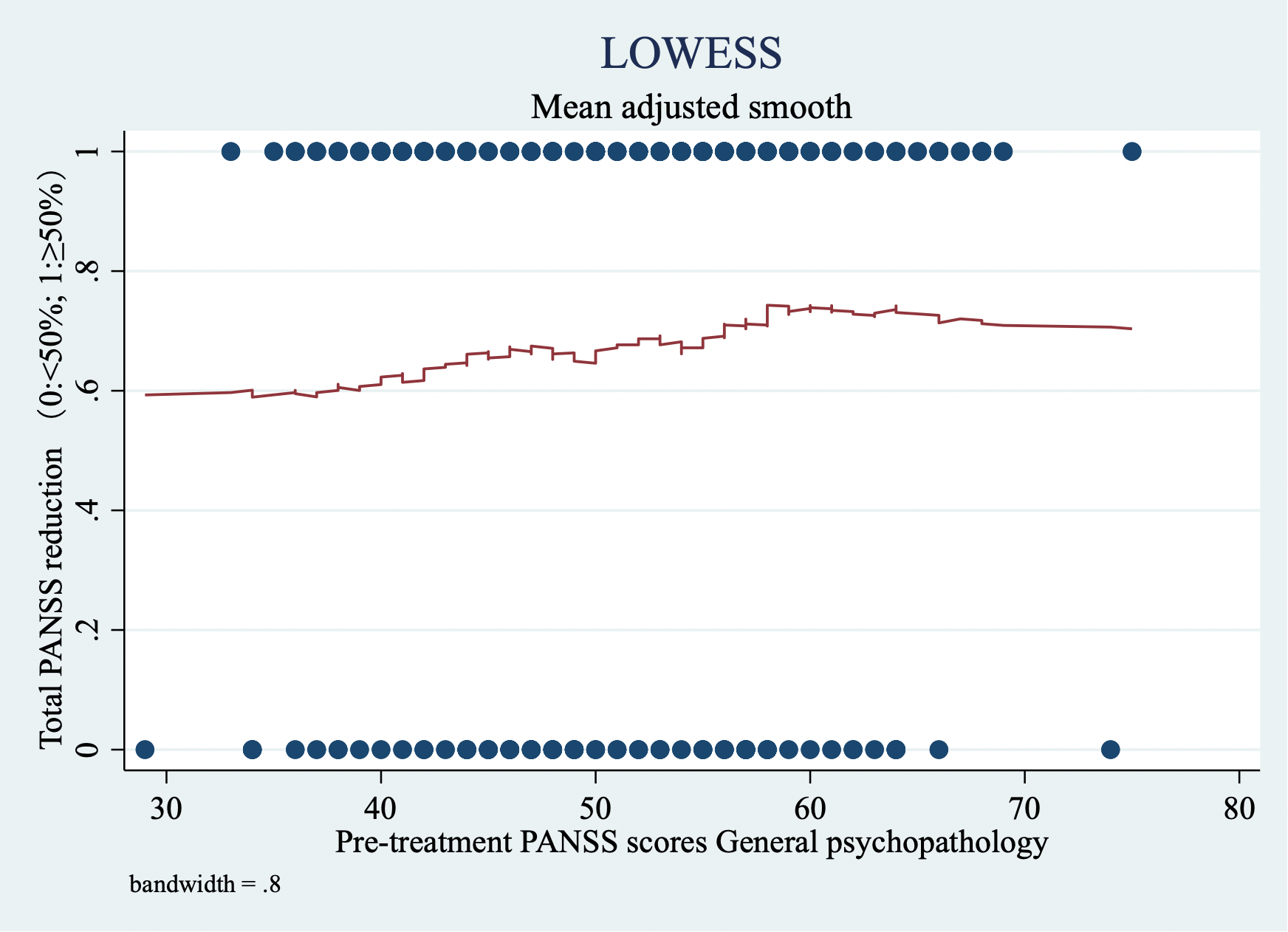

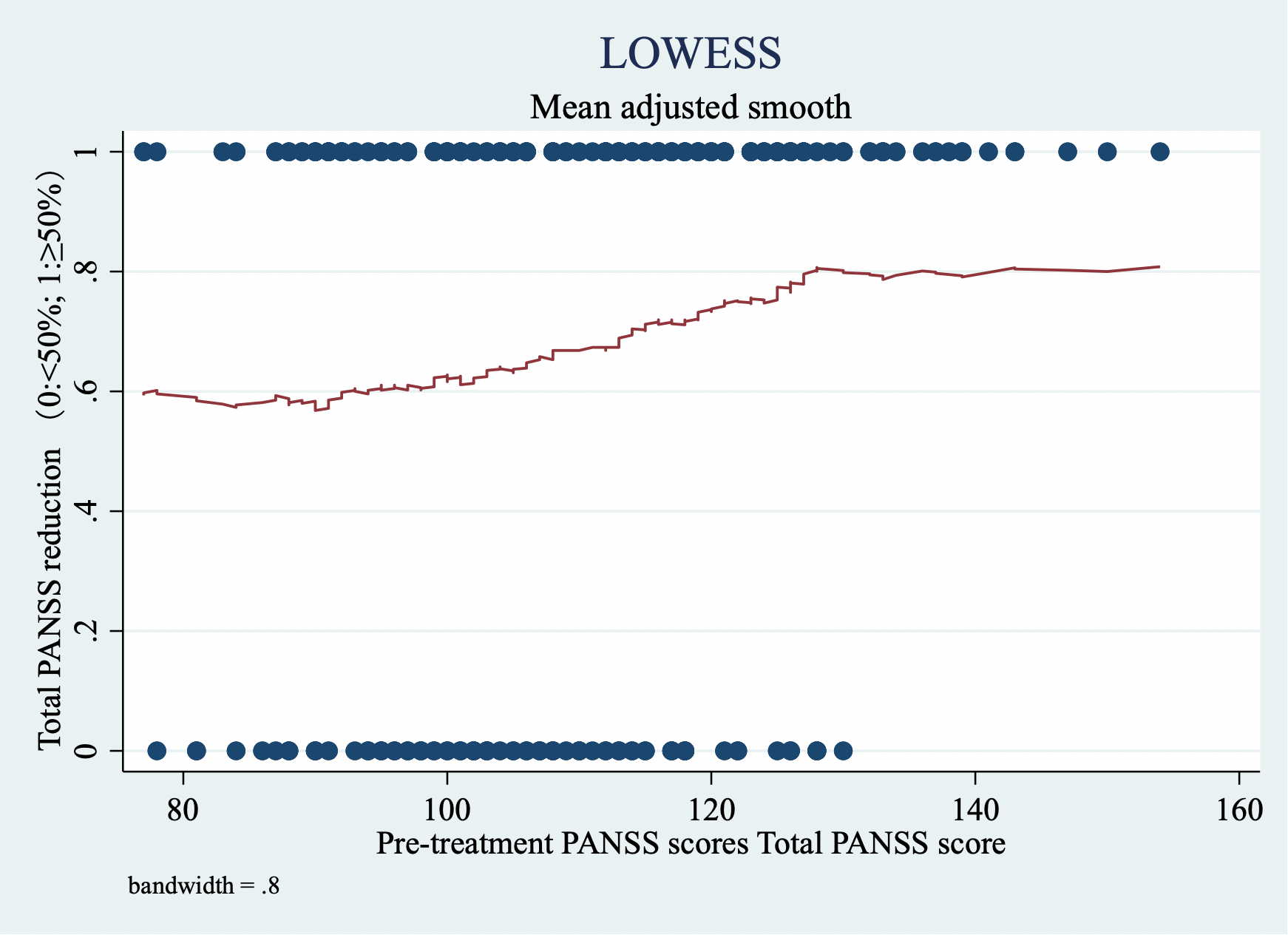


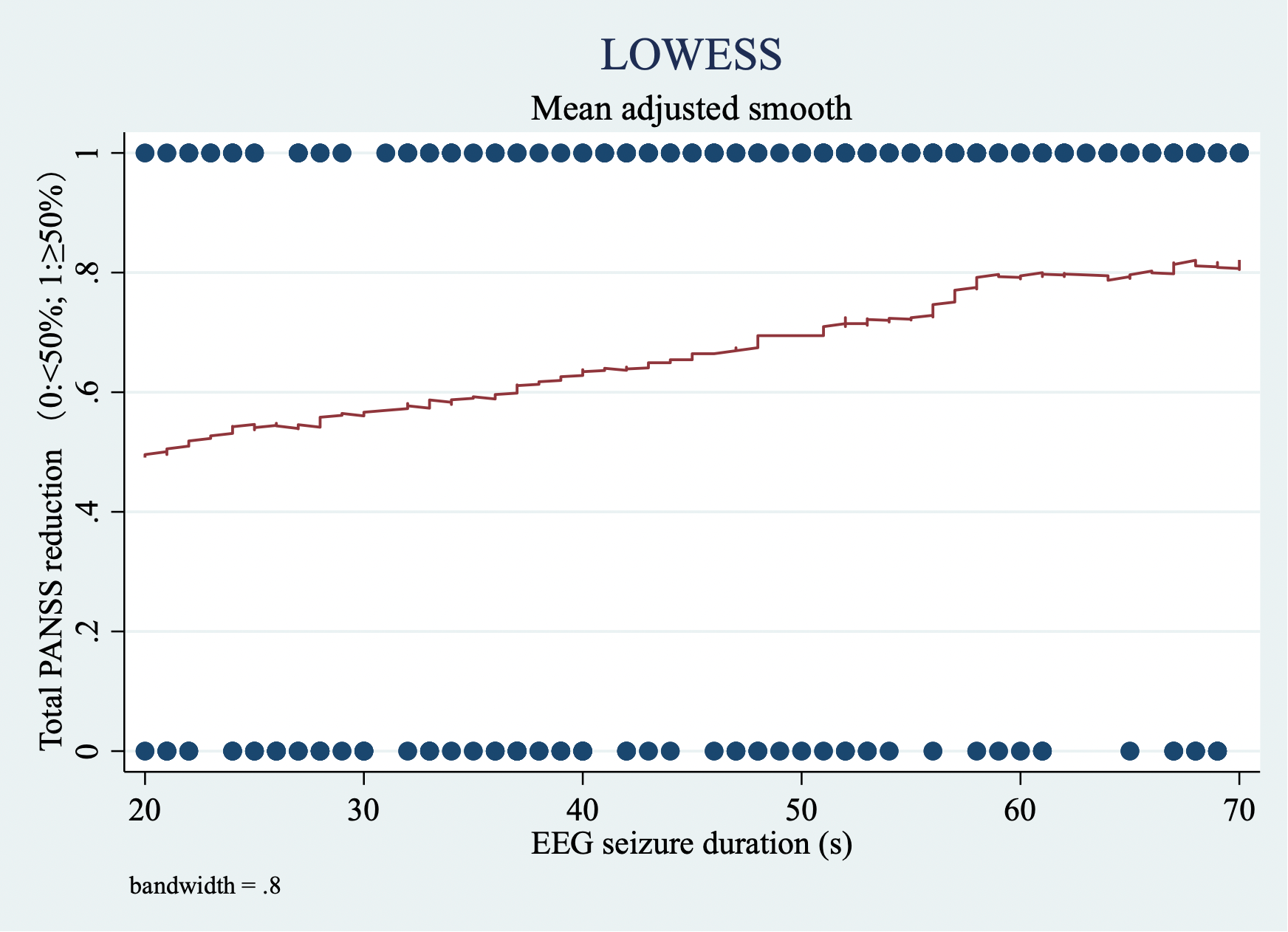

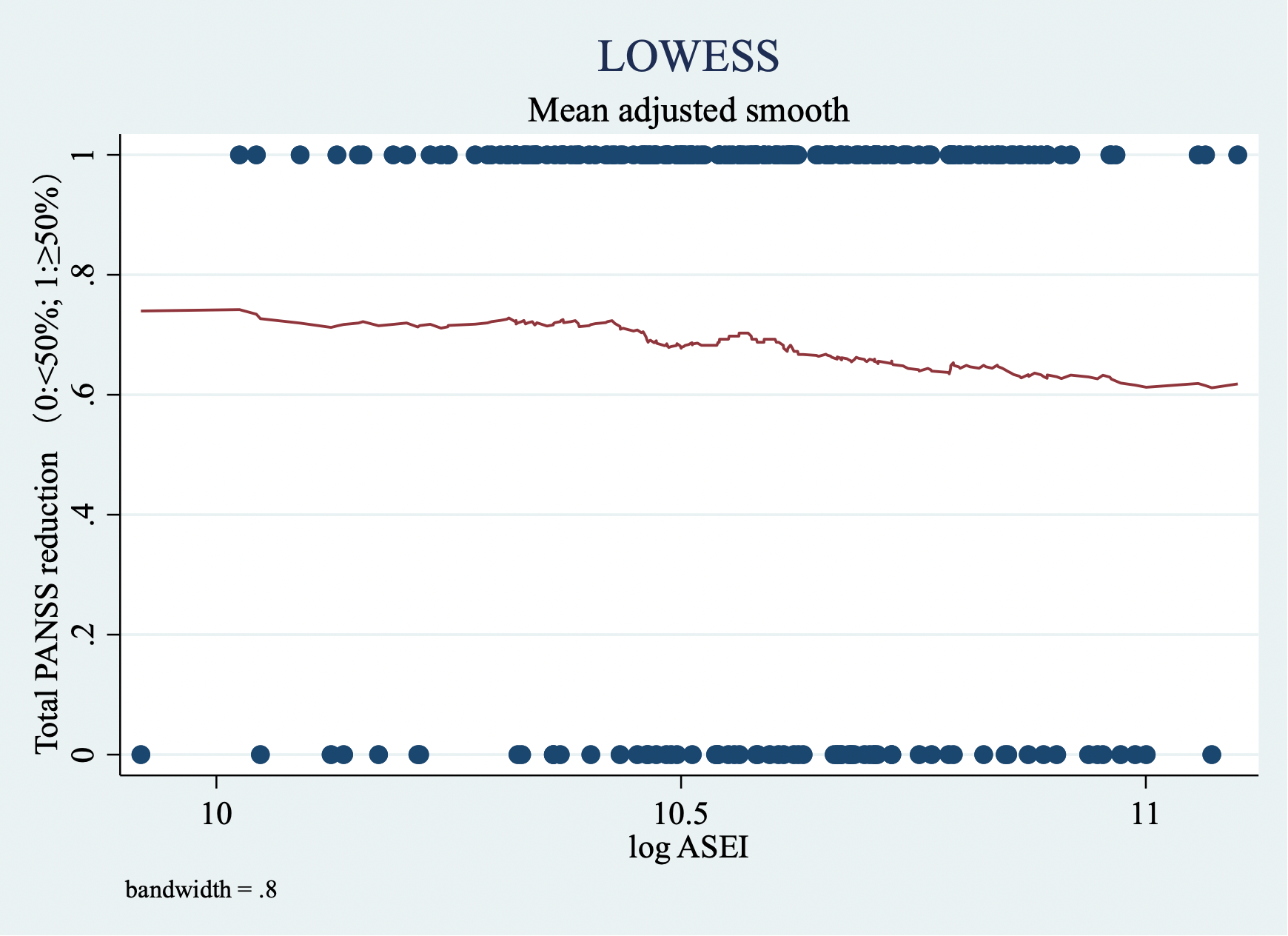


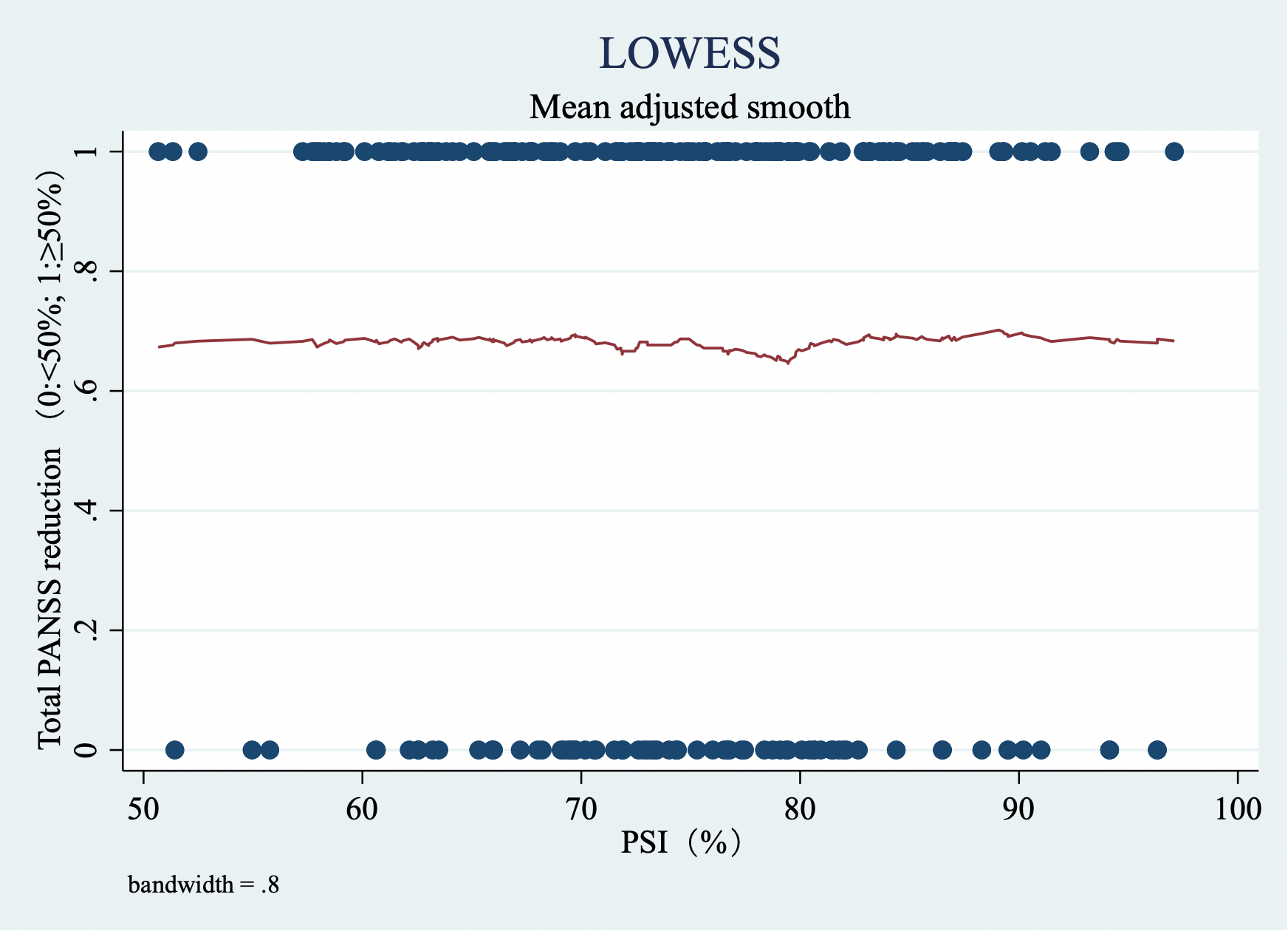

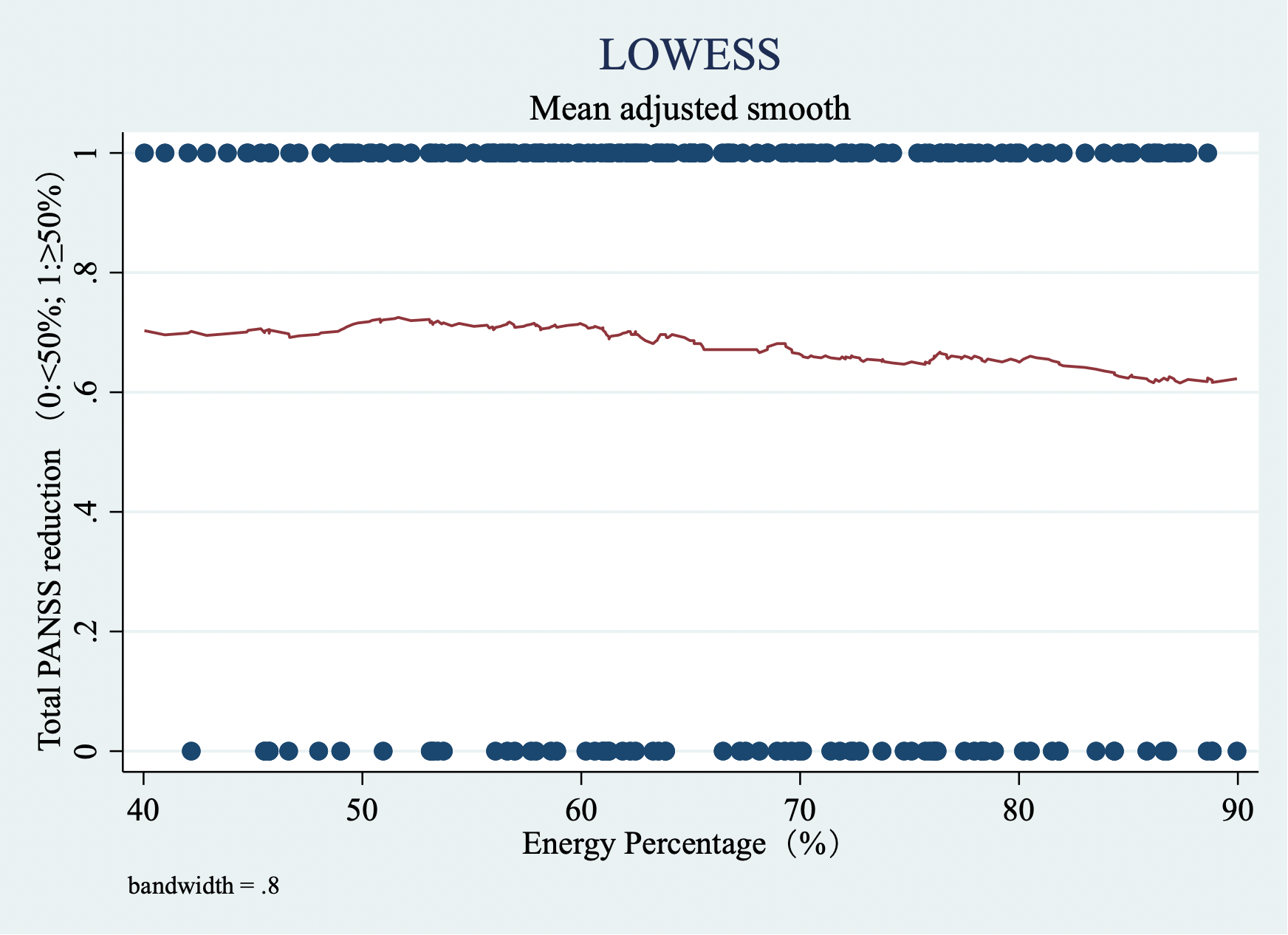


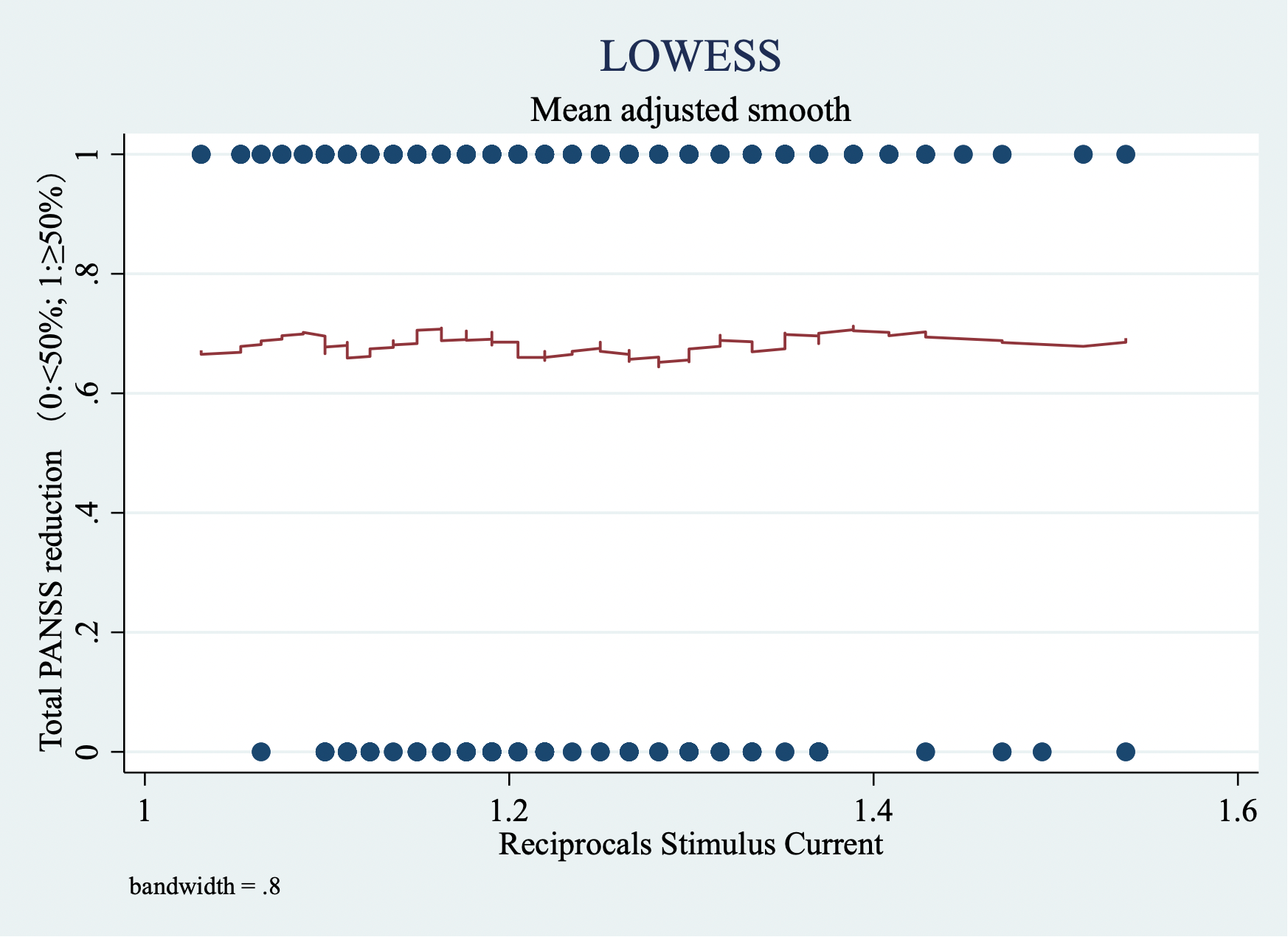

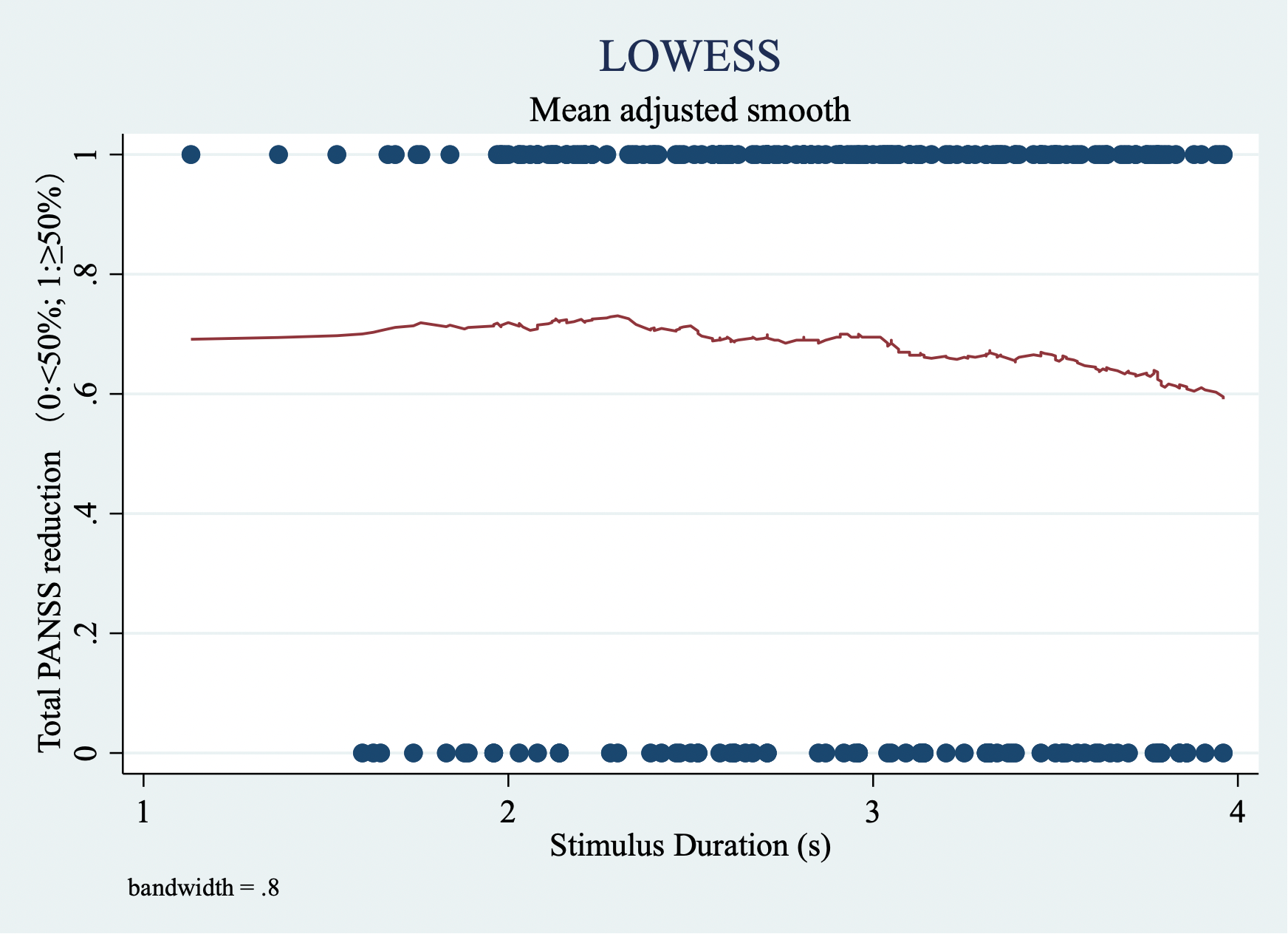


**Supplementary materials 7 the effect sizes Cohen’s d**

We calculated effect sizes (Cohen’s d) with 95% confidence intervals. The effect size estimates yielded conclusions consistent with our original p-value–based findings, supporting the robustness of the observed associations.

|  | Skewness | Kurtosis | p-value | Cohen’s d | 95% CI | |
| --- | --- | --- | --- | --- | --- | --- |
| BMI | 0.690 | 0.069 | 0.174 | -0.144 | -0.412 | 0.124 |
| Antipsychotic dose | 0.007 | 0.506 | 0.025 | 0.043 | -0.224 | 0.311 |
| **MECT Parameters** | | | | | | |
| EEG seizure duration | 0.203 | 0.000 | 0.000 | -0.648 | -0.921 | -0.374 |
| ASEI | 0.012 | 0.939 | 0.046 | 0.204 | -0.065 | 0.472 |
| PSI | 0.828 | 0.108 | 0.265 | 0.079 | -0.189 | 0.347 |
| Static Impedance (Ω) | 0.600 | 0.000 | 0.000 | -0.028 | -0.296 | 0.239 |
| Dynamic Impedance (Ω) | 0.930 | 0.000 | 0.000 | 0.274 | 0.005 | 0.543 |
| Energy Percentage (%) | 0.965 | 0.000 | 0.001 | 0.217 | -0.052 | 0.485 |
| Stimulus Charge (mC) | 0.878 | 0.000 | 0.000 | 0.016 | -0.251 | 0.284 |
| Stimulus Current (A) | 0.775 | 0.108 | 0.260 | -0.072 | -0.339 | 0.196 |
| Stimulus Frequency (Hz) | 0.213 | 0.000 | 0.000 | 0.148 | -0.120 | 0.415 |
| Stimulus Duration (s) | 0.052 | 0.000 | 0.001 | 0.144 | -0.124 | 0.412 |
| **Pre-treatment PANSS scores** | | | | | | |
| Negative symptoms | 0.152 | 0.000 | 0.000 | -0.991 | -1.271 | -0.708 |
| Positive symptoms | 0.000 | 0.033 | 0.000 | -0.169 | -0.437 | 0.099 |
| General psychopathology | 0.346 | 0.211 | 0.290 | -0.282 | -0.551 | -0.013 |
| Total PANSS score | 0.477 | 0.246 | 0.393 | -0.503 | -0.774 | -0.231 |

**Supplementary materials 8 The** ROC, AUC, and model calibration evaluated using bootstrap validation.
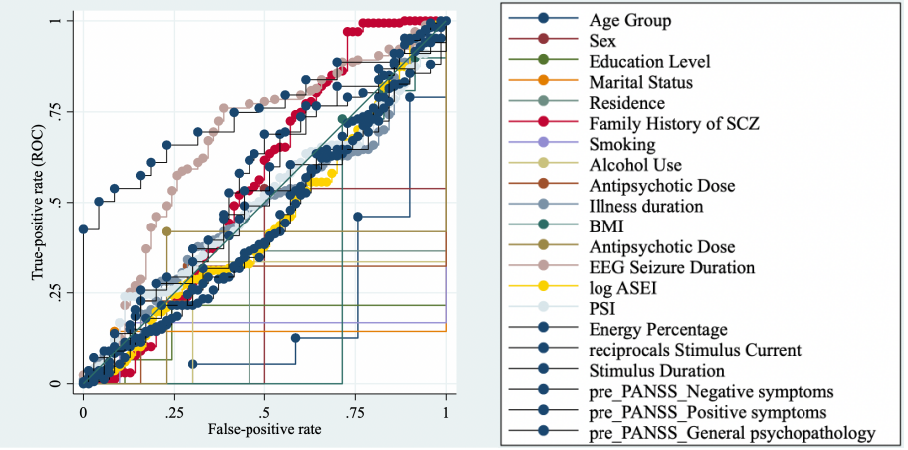

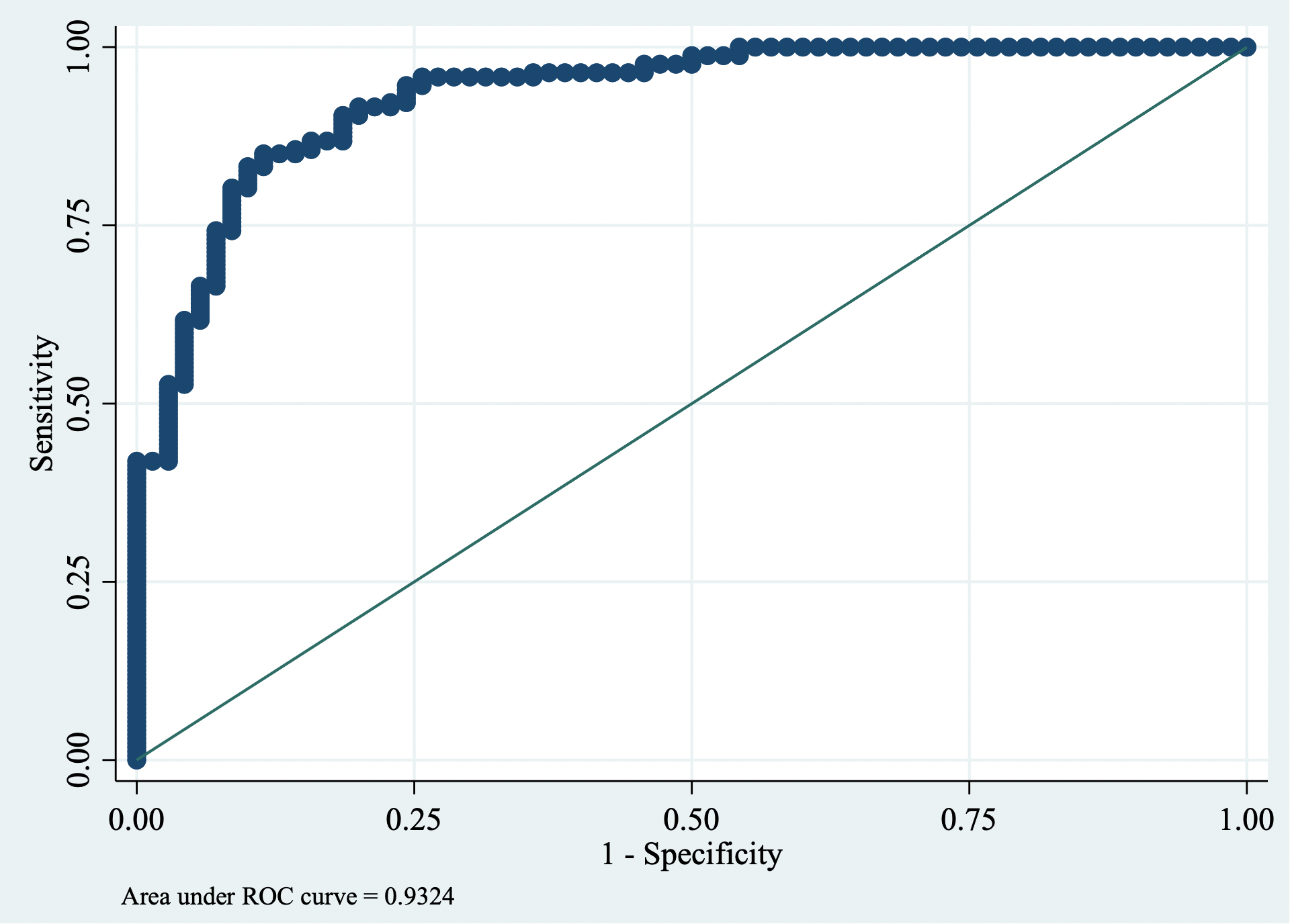


**Supplementary materials 9 Partial effect plots for continuous predictors**

1. **Age: older age was a negative predictor**


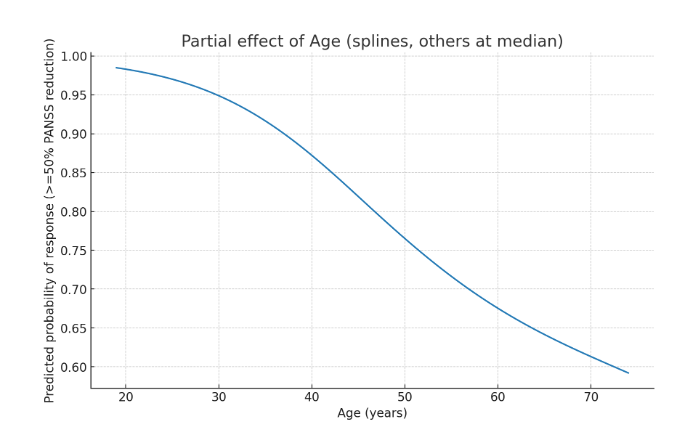


1. **Illness duration: the longer was a negative predictor**


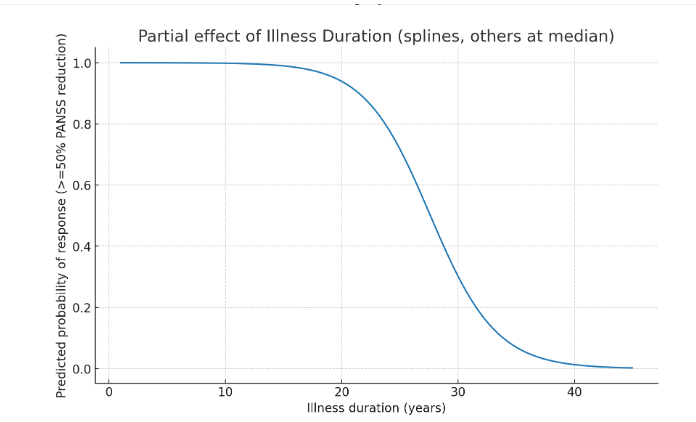

Supplement: Supplementary file 1 [file Supplementaryfile1.docx]
